# Inflammasomes in neuroinflammatory and neurodegenerative diseases

**DOI:** 10.15252/emmm.201810248

**Published:** 2019-04-23

**Authors:** Sofie Voet, Sahana Srinivasan, Mohamed Lamkanfi, Geert van Loo

**Affiliations:** ^1^ VIB Center for Inflammation Research Ghent Belgium; ^2^ Department of Biomedical Molecular Biology Ghent University Ghent Belgium; ^3^ Department of Internal Medicine Ghent University Ghent Belgium; ^4^ Janssen Immunosciences, World without Disease Accelerator, Pharmaceutical Companies of Johnson & Johnson Beerse Belgium

**Keywords:** disease, inflammasome, inflammation, microglia, neurodegeneration, Immunology, Neuroscience

## Abstract

Neuroinflammation and neurodegeneration often result from the aberrant deposition of aggregated host proteins, including amyloid‐β, α‐synuclein, and prions, that can activate inflammasomes. Inflammasomes function as intracellular sensors of both microbial pathogens and foreign as well as host‐derived danger signals. Upon activation, they induce an innate immune response by secreting the inflammatory cytokines interleukin (IL)‐1β and IL‐18, and additionally by inducing pyroptosis, a lytic cell death mode that releases additional inflammatory mediators. Microglia are the prominent innate immune cells in the brain for inflammasome activation. However, additional CNS‐resident cell types including astrocytes and neurons, as well as infiltrating myeloid cells from the periphery, express and activate inflammasomes. In this review, we will discuss current understanding of the role of inflammasomes in common degenerative diseases of the brain and highlight inflammasome‐targeted strategies that may potentially treat these diseases.

**Glossary**
Blood–brain barrier (BBB): a physiological barrier between the blood and the CNS parenchyma. The BBB is formed by endothelial cells that are joined by complex tight junctions and coated by a basement membrane and an additional membrane of astrocytic end‐feet, known as the glia limitans.
Caspases: a family of cysteine‐dependent aspartate specific proteases that play a central role in inflammation and programmed cell death.
Cerebrospinal fluid (CSF): contained in the ventricles of the brain and the cranial and spinal subarachnoid spaces. It provides a mean for transporting different molecules, including cytokines, neurotransmitters, and hormones, but also resides innate immune cells.
CNS macrophages: non‐parenchymal macrophages of the CNS that are located in meninges, choroid plexus, and perivascular spaces.
Creutzfeldt–Jakob disease: fatal degenerative prion disease that can be sporadic, hereditary, or acquired. The acquired form of the disease is caused by exposure to the misfolded scrapie form of the PrP protein.
Cuprizone‐induced demyelination: an experimental model to study local demyelination of the corpus callosum, which is induced by the administration of the copper chelator cuprizone (bis(cyclohexanone)oxaldihydrazone) to the food of mice during 5 weeks.
Dopaminergic neurons: the main dopamine‐producing cells of the CNS and essential for controlling key functions of the brain, including voluntary movement, reward processing, mood, and working memory.
Encephalomyelitis: general term to describe inflammation in the brain or spinal cord. Autoimmune encephalomyelitis is caused by an abnormal immune response to a self‐antigen.
Experimental autoimmune encephalomyelitis (EAE): the main rodent model of MS. EAE is actively induced by peripheral immunization with myelin‐specific proteins or peptides in combination with an adjuvant, or passively by transfer of encephalitogenic T cells.
Familial Mediterranean Fever: the most common monogenic autoinflammatory disease and is characterized by periodic fevers with childhood onset, and frequently associated with serositis and joint pain. It predominantly affects Mediterranean populations.
Grand‐mal seizures: or tonic‐clonic seizures: seizures that are characterized by the loss of consciousness and violent muscle contractions. This seizure comes in two phases: a tonic phase followed by a clonic phase. The brief tonic phase features loss of consciousness and muscle stiffening, while in the clonic phase the muscles go into rhythmic contractions.
Induced pluripotent stem cells (iPSCs): generated by genetic reprogramming of adult somatic cells. The advantage over other types of stem cells is that they are not derived from a human embryo.
Ischemic infarct: an infarct caused by the interruption of the blood flow in the brain due to occlusion of a cerebral vessel.
Middle cerebral artery occlusion: experimental stroke model that involves the permanent or transient occlusion of the middle cerebral artery. The middle cerebral artery is one of the three arteries that supply blood to the cerebrum. In the “filament model”, a suture filament is (transiently) introduced into the internal carotid artery and forwarded until the tip occludes the middle cerebral artery.
Neonatal‐onset multisystem inflammatory disease (NOMID): or chronic infantile neurologic cutaneous and articular (CINCA): the most severe cryopyrin‐associated periodic syndrome (CAPS). NOMID is characterized by neonatal‐onset skin lesions, chronic aseptic meningitis, and recurrent fever along with joint symptoms.
Sepsis: a life‐threatening systemic inflammatory response syndrome (SIRS) caused by the body's response to an infection.
Spatial memory: an important cognitive function that allows us to recall three‐dimensional objects or places.
Steatohepatitis: a type of fatty liver disease and is characterized by the accumulation of lipids and the infiltration of inflammatory cells in the hepatic parenchyma.
Striata: dopamine neurons from the substantia nigra pars compacta project to the caudate and the putamen of the basal ganglia, together referred to as the striatum.
Substantia nigra pars compacta: structure in the brainstem that contains most of the dopamine‐producing neurons.

## Introduction

The innate immune system is a rapid and coordinated cellular defense response that aims to eliminate the threat posed by both sterile and infectious insults. Recognition of these pathogenic agents is mediated by pattern recognition receptors (PRRs) that sense pathogen‐associated molecular patterns (PAMPs) and host‐ or environment‐derived danger‐associated molecular patterns (DAMPs). In the central nervous system (CNS), these PRRs are primarily expressed by microglia, astrocytes, and macrophages, but also oligodendrocytes, neurons, and endothelial cells express a repertoire of PRRs (Lampron *et al*, 2013; Walsh *et al*, 2014). PRRs can either be membrane‐bound, as is the case for the Toll‐like receptors (TLRs), to sense signals in the extracellular environment or in the endosome, or they can be intracellular, as with the nucleotide‐binding domain and leucine‐rich repeat‐containing receptors (NLRs) and AIM2‐like receptors (ALRs). An important subgroup of cytosolic PRRs that includes members of the NLR and ALR families as well as the tripartite motif (TRIM) family member pyrin critically contributes to the innate immune response by assembling so‐called “inflammasomes”.

## General concepts of inflammasome biology

**Figure 1:**
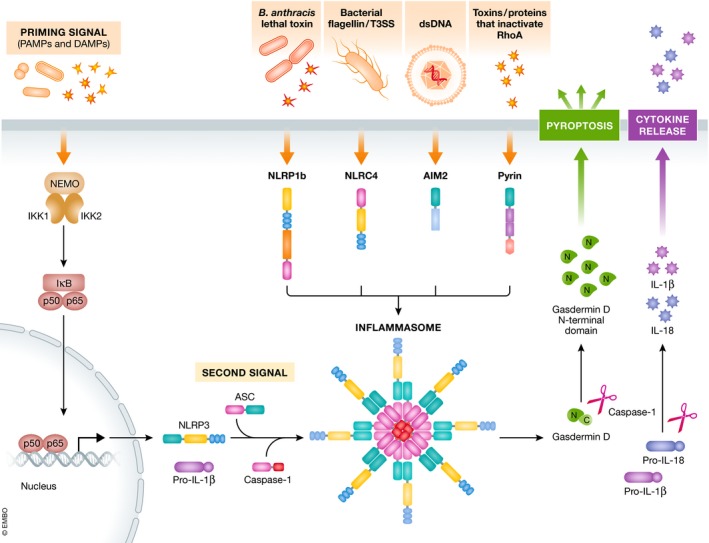
Inflammasome activation and signaling Inflammasomes assemble in a stimulus‐specific manner. Different DAMPs and PAMPs are able to induce NLRP3, while NLRP1b responds to Bacillus anthracis lethal toxin, NLRC4 recognizes bacterial flagellin and/or the type III secretion system of bacterial pathogens, AIM2 is specifically activated by dsDNA, and pyrin recognizes the inactivation of RhoA by toxins and effector proteins. Activation of the NLRP3 inflammasome involves a two‐step mechanism. The priming signal is detected by membrane‐bound PRRs, including TLRs and C‐type lectin receptors (CLRs) and induces NF‐κB‐dependent transcription of NLRP3 and pro‐IL‐1β precursor protein, and controls post‐translational modifications that license NLRP3 activation. The second activation signal is necessary for inflammasome formation, depending on the oligomerization and subsequent activation of procaspase‐1. Active caspase‐1 then cleaves pro‐IL‐1β and pro‐IL‐18 to their mature forms IL‐1β and IL‐18 which get secreted. In addition, caspase‐1 can cleave gasdermin D, releasing its N‐terminal fragment which translocates to the plasma membrane inducing pore formation and pyroptotic cell death. In contrast to NLRP3, other inflammasome receptors do not need this initial priming signal to induce inflammasome activation and cytokine release.

Inflammasomes are cytosolic multiprotein complexes which upon assembly activate the pro‐inflammatory caspase‐1 (see Glossary) that is responsible for the maturation and secretion of the inflammatory cytokines IL‐1β and IL‐18, and additionally induce pyroptosis (Lamkanfi & Dixit, 2014; Broz & Dixit, 2016). Briefly, inflammasome‐inducing stimuli trigger the oligomerization of PRR proteins and the recruitment of pre‐existing procaspase‐1 zymogens into the complex, leading to their proximity‐induced autoactivation to generate active caspase‐1. Consequently, caspase‐1 will cleave the biologically inactive pro‐peptides pro‐IL‐1β and pro‐IL‐18 into mature cytokines which are then secreted by the cell. Next to its role in the maturation of pro‐IL‐1β and pro‐IL‐18, caspase‐1 can also induce a pro‐inflammatory form of cell death, pyroptosis, that features early plasma membrane rupture, thereby releasing the soluble intracellular fraction that fuels the inflammatory response (Lamkanfi & Dixit, 2012, 2014). Central in this process is the executioner protein gasdermin D (GSDMD)—a substrate of murine caspase‐1 and caspase‐11, and human caspase‐1, caspase‐4, and caspase‐5—the amino‐terminal domain of which upon cleavage oligomerizes and perforates the plasma membrane to induce cell swelling and osmotic lysis (Shi *et al*, 2017) (Fig 1).

**Figure 2:**
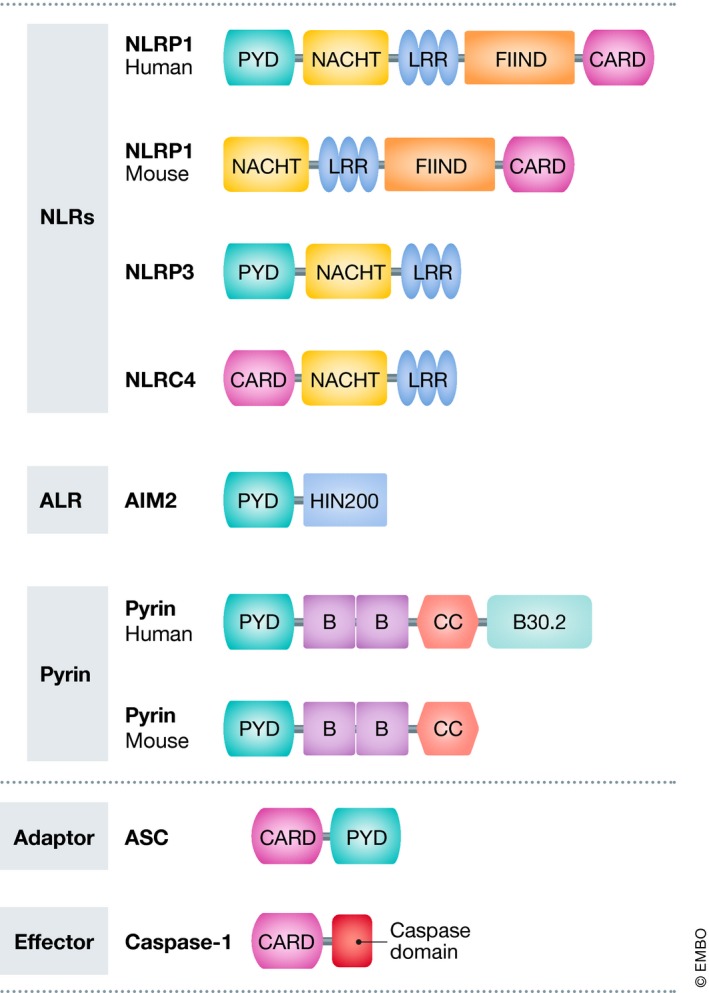
Domain structure of inflammasomes A subset of NLRs and ALRs can trigger the formation of inflammasomes. NLR family members have a nucleotide‐binding and oligomerization domain (NACHT/NBD), as well as leucine‐rich repeat (LRR) motifs, typically located in the center and carboxy terminus of the NLR proteins, respectively. The NACHT motif is usually flanked by an additional amino‐terminal domain, either CARD or PYD, and these domains are used for further sub‐classification of inflammasomes. These domains allow the recruitment of adaptor and effector proteins to the inflammasome signaling complex. The NLR gene family consists of 22 human members and 34 murine members, many of which the function is not always clear (Lamkanfi & Dixit, 2012; Broz & Dixit, 2016). In addition to the NLR‐containing inflammasomes, the ALR family member AIM2 can also assemble an inflammasome complex. AIM2 is characterized by an amino‐terminal PYD domain and one or two DNA‐binding HIN200 domains (Hornung *et al*, 2009). Pyrin, also known as TRIM20, features a PYD domain, two B‐boxes, and a coiled‐coil domain, whereas the human pyrin also has an additional C‐terminal B30.2 domain. ASC is the critical adaptor protein for many inflammasome complexes and is composed of CARD and PYD domains, the latter being necessary for homotypic interaction with a PYD‐containing inflammasome sensor (NLRP3, AIM2). Procaspase‐1 features a CARD domain, in addition to its caspase domain, and homotypic CARD interactions result in direct or indirect (via ASC) recruitment of procaspase‐1 to the inflammasome complex. Inflammasome activation involves ASC and procaspase‐1 recruitment, resulting in ASC oligomerization into a macromolecular aggregate, known as an ASC speck (Broz & Dixit, 2016).

One way to classify inflammasome complexes is based on the receptor that initiates signaling (Lamkanfi & Dixit, 2012). The core inflammasome components consist of the cytosolic NLR, ALR, and pyrin receptors, the adaptor apoptosis‐associated speck‐like protein containing a caspase recruitment domain (ASC) and procaspase‐1. ASC is composed of a pyrin domain (PYD) and a caspase recruitment domain (CARD) and functions as an adaptor that links the PYD of the NLR or pyrin and the CARD of procaspase‐1 (Fig 2). Some inflammasomes, such as NLRC4 and NLRP1b, do not require ASC and may directly recruit procaspase‐1 through their respective CARDs, although under wild‐type conditions ASC does contribute to optimal caspase‐1 activation and efficient secretion of IL‐1β and IL‐18 (Broz *et al*, 2010; Guey *et al*, 2014; Van Opdenbosch *et al*, 2014). Assembly and activation of inflammasomes requires detection of specific signals: Murine Nlrp1b responds to *Bacillus anthracis* lethal toxin (LeTx) (Boyden & Dietrich, 2006); NLRC4 detects intracellular flagellin and components of type III secretion systems (T3SS) of bacterial pathogens (Franchi *et al*, 2006; Miao *et al*, 2006, 2010); AIM2 physically binds cytosolic double‐stranded DNA (dsDNA) (Hornung *et al*, 2009); and pyrin indirectly responds to toxins that covalently inactivate the small GTPase RhoA (Xu *et al*, 2014). NLRP3, the most widely studied inflammasome, responds to a broad spectrum of activating stimuli that includes a suite of bacterial, fungal, and viral PAMPs, DAMPs such as ATP and uric acid crystals, and crystalline and aggregated substances such as asbestos, silica, and amyloid‐β fibrils (Lamkanfi & Dixit, 2012). NLRP3 activation is unique in the sense that it involves a two‐step process. A first signal, or priming signal, results in the NF‐κB‐dependent transcriptional upregulation of NLRP3 and pro‐IL‐1β, but also controls post‐translational modifications of NLRP3 as highlighted by the mapped ubiquitin and phosphorylation sites on NLRP3 (Yang *et al*, 2017). This is followed by a second “activation” signal that induces the oligomerization and activation of the NLRP3 inflammasome (Fig 1). Besides canonical NLRP3 inflammasome signaling, the non‐canonical NLRP3 inflammasome pathway involves activation of caspase‐11 (or its human orthologs caspase‐4 and caspase‐5) by cytosolic LPS, and the induction of pyroptosis through the cleavage of GSDMD and the release of high mobility group box 1 protein (HMGB1) and IL‐1α (Lamkanfi & Dixit, 2014; Shi *et al*, 2017). Apart from AIM2 and the NAIP/NLRC4 complexes, the common secondary messengers or physical ligands that bind and trigger inflammasome assembly await discovery.

## Inflammasomes and the central nervous system

IL‐1β and IL‐18 have important functions in the CNS, and many cell types in the brain express their cognate receptors that initiate inflammatory signaling cascades that may contribute to neuronal injury and cell death (Allan *et al*, 2005; Alboni *et al*, 2010). Hence, increased levels of IL‐1β and IL‐18 are often observed upon CNS infection, brain injury, and neurodegenerative diseases (Heneka *et al*, 2014, 2018). IL‐1β and IL‐18 are also important for physiological functions in the CNS and have been shown to participate to processes of cognition, learning, and memory (Tsai, 2017). Pyroptosis additionally contributes to inflammasome‐driven pathology through the release of other inflammatory mediators and DAMPs. Apoptosis and necroptosis are additional cell death modes that have been shown to promote neuroinflammation and neuronal degeneration in several neurodegenerative pathologies, including multiple sclerosis, amyotrophic lateral sclerosis, Parkinson's disease, and Alzheimer's disease (Zhang *et al*, 2017; Yuan *et al*, 2019).

**Figure 3:**
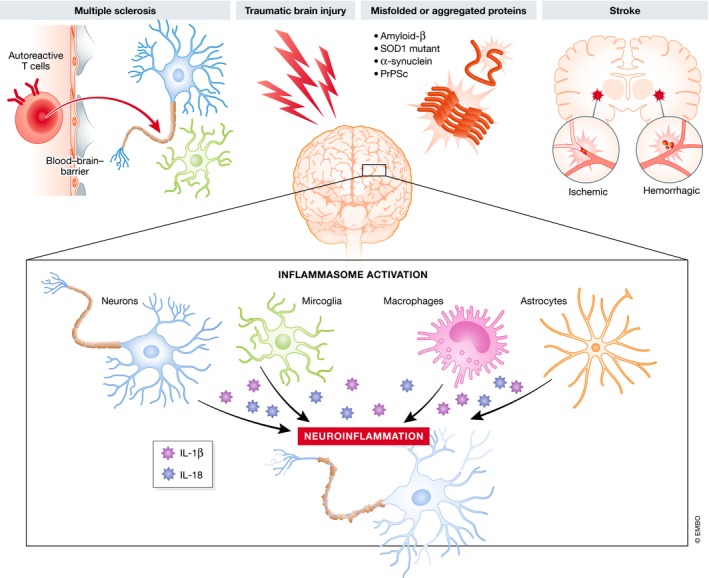
Inflammasome activation in neurodegenerative disease Inflammasomes can be activated in the CNS in response to acute injury (traumatic brain injury and stroke), autoimmune‐mediated injury (multiple sclerosis), and accumulation of misfolded or aggregated proteins in the brain (Alzheimer's disease, amyotrophic lateral sclerosis, Parkinson's disease, and prion disease). Inflammasome activation has been demonstrated in CNS‐resident cell types, including microglia, astrocytes, and neurons, but also in CNS‐infiltrating cells, such as in infiltrating macrophages. Although most research on neurodegenerative diseases has focused on the importance of the NLRP3 inflammasome, also other inflammasome types can be activated in the brain and have been demonstrated in neurodegenerative disorders. Overall, inflammasome activation results in caspase‐1‐mediated cleavage of pro‐IL‐1β and pro‐IL‐18, and the subsequent release of the mature cytokines. High levels of IL‐1β and IL‐18 can be detected in many neurodegenerative conditions and are considered to be crucial for the establishment of a chronic inflammatory environment, leading to neuronal dysfunction and eventually neurodegeneration.

Although inflammasome signaling in the CNS is mainly attributed to microglia, the key innate immune cells of the brain, expression of inflammasome components has also been reported in other cell types of the CNS, including neurons (Kaushal *et al*, 2015), astrocytes (Freeman *et al*, 2017), perivascular CNS macrophages (Kawana *et al*, 2013), oligodendrocytes (Mckenzie *et al*, 2018), and endothelial cells (Gong *et al*, 2018) (Fig 3). However, current understanding of inflammasome activation in microglia and its role in CNS inflammation and disease is still fragmentary and primarily based on *in vitro* studies with primary microglia and microglial cell lines, and *in vivo* studies with transgenic knockout mice that lack expression of specific inflammasome components throughout the body. More appropriate research tools that allow CNS‐specific inflammasome targeting are needed to investigate the relative contribution of local inflammasome signaling in specific brain cell types to overall CNS pathology. Furthermore, evidence for inflammasome activation in patients with neurodegenerative disease often relies on detection of higher IL‐1β transcript levels and/or increased gene and protein expression of inflammasome components. However, increased expression levels of these NF‐κB responsive genes are largely indicative of an ongoing inflammatory response rather than direct support for inflammasome engagement. We here review the current knowledge on the relevance of inflammasome activation for the most common neurodegenerative pathologies (Fig 3 and Table 1) and highlight emerging strategies for the treatment of inflammasome‐driven diseases. Although inflammasome activation is also central to the pathology of many CNS infectious diseases, such as Zika, HIV, and West Nile virus, and bacterial infections that induce meningitis (Walsh *et al*, 2014; Mamik & Power, 2017; Heneka *et al*, 2018), these conditions are outside the scope of this review.

**Table 1:** Overview of the described neurodegenerative disease models

Disease model	Description
**Multiple sclerosis**	
EAE	EAE is actively induced by peripheral immunization with myelin‐specific proteins or peptides in combination with an adjuvant, or passively by transfer of encephalitogenic T cells
Cuprizone	Administration of the copper chelator cuprizone will induce local demyelination of the corpus callosum
**Stroke**	
Permanent MCAO	Permanent occlusion of the middle cerebral artery is obtained using an intraluminal suture
Transient MCAO	Intraluminal suture MCAO utilizes a suture inserted into the middle cerebral artery to interrupt the blood flow for a specific duration and is afterward removed. Embolic stroke MCAO uses an autologous blood clot injected into the MCA to occlude the vessel
Intracerebral hemorrhage (ICH)	Stroke condition provoked by injection of autologous arterial blood into the basal ganglia
**TBI and SCI**	
Controlled cortical impact (CCI)	A mechanical model of traumatic brain injury. Following craniotomy, the CCI device mechanically transfers energy onto the dura mater damaging the cortex, and sometimes the subcortical structures
Impact acceleration model	The exposed skull is covered with a steel disk and a weight is dropped onto the steel disk
Contusion model of spinal cord injury	A transient force is applied by either an electromagnetic device or a weight‐drop to displace and damage the spinal cord
**Alzheimer disease**	
APP/PS1	Mouse model expressing human APP695 with the Swedish double mutation (K670N/M671L) and a mutant human presenilin 1 (PS1‐dE9)
3xTgAD	Mouse model expressing human APP695 with the Swedish double mutation (K670N/M671L), human PS1 with the M146V mutation, and human Tau with the P301L mutation
Tg2567	Mouse model expressing human APP695 with the Swedish double mutation (K670N/M671L) at the β‐secretase cleavage site
5xFAD	Mouse model expressing human APP and PSEN1 transgenes with a total of five AD‐linked mutations: the Swedish (K670N/M671L), Florida (I716V), and London (V717I) mutations in APP, and M146L and L286V mutations in PSEN1
**Amyotrophic lateral sclerosis**	
TgSOD1‐G93A	Mouse model expressing a G93A mutant form of human SOD1
hSOD1 G37R	Mouse model expressing a G37R mutant form of human SOD1
hSOD1 G85R	Mouse model expressing a G85R mutant form of human SOD1
**Parkinson disease**	
A53T	Mouse model expressing the mutant human A53T alpha‐synuclein.
LPS‐induced PD	Injection of LPS in the left substantia nigra pars compacta
6‐hydroxydopamine‐induced PD	Injection of 6‐hydroxydopamine in the medial forebrain bundle
MPTP‐induced PD	Intraperitoneal injection of MPTP five times at 2‐h intervals
**Prion disease**	
Scrapie‐infected	Intracerebral infections with brain homogenate of scrapie strain 139A‐infected mice or intracerebral injection with RML6 (passage 6 of Rocky Mountain Laboratory strain mouse‐adapted scrapie prions)
Tg(CJD)	Mouse model expressing a misfolded mutant PrP (D177N/V128)
**Huntington disease**	
R6/2	Transgenic mice expressing exon 1 of human huntingtin with expanded CAG/polyglutamine repeat

## Inflammasome activation in multiple sclerosis and experimental autoimmune encephalomyelitis

Multiple sclerosis (MS) is the most common chronic inflammatory disease of the CNS. MS is characterized by a compromised blood–brain barrier (BBB) that leads to immune cell infiltration from the periphery and local microglia and astrocyte activation, which together promote inflammation, demyelination, and neurodegeneration (Baecher‐Allan *et al*, 2018). Experimental autoimmune encephalomyelitis (EAE) is a widely used rodent model of MS (Ransohoff, 2012), and both IL‐1β and IL‐18 were shown to critically contribute to EAE (Sutton *et al*, 2006; Gris *et al*, 2010; Lévesque *et al*, 2016). Most studies on the involvement of inflammasomes in MS have focused on the peripheral immune response that is shaped by lymphocytes and macrophages that enter the CNS during MS pathology. Caspase‐1, IL‐18, and IL‐1β are upregulated in peripheral blood mononuclear cells (PBMCs) and cerebrospinal fluid (CSF) of MS patients, as are levels of the NLRP3 inflammasome‐activating DAMPs ATP and uric acid (Inoue & Shinohara, 2013; Mamik & Power, 2017). Moreover, caspase‐1 expression was shown to be elevated in acute and chronic demyelinating lesions (Voet *et al*, 2018), and caspase‐1 and ASC have recently been proposed as candidate biomarkers for MS onset (Keane *et al*, 2018).

The role of the NLRP3 inflammasome in EAE‐associated T‐cell priming and trafficking into the CNS is supported by the analysis of NLRP3, caspase‐1, and ASC knockout mice (Furlan *et al*, 1999; Gris *et al*, 2010; Inoue *et al*, 2012a). Moreover, the pharmacological NLRP3 inflammasome inhibitor MCC950/CRID3 was shown to suppress IL‐1β production and attenuate EAE severity in wild‐type mice (Coll *et al*, 2015). Similarly, pharmacological blockade of caspase protease activity with Z‐Val‐Ala‐DL‐Asp‐fluoromethylketone or the inflammatory caspase prodrug VX‐765 reduced EAE symptoms in mice (Furlan *et al*, 1999; Mckenzie *et al*, 2018). Interestingly, IFNβ, the first line treatment for MS, is only effective in an NLRP3 inflammasome‐dependent EAE subtype, whereas NLRP3‐independent EAE could not be reversed by IFN‐β therapy (Inoue *et al*, 2012b, 2016). We recently provided direct genetic evidence for the relevance of inflammasome signaling in microglia and border‐associated macrophages during EAE (Voet *et al*, 2018). The anti‐inflammatory protein A20 has been shown to negatively regulate NLRP3 inflammasome activation (Vande Walle *et al*, 2014), and deletion of A20 in microglia and CNS macrophages exacerbated EAE in mice due to NLRP3 hyperactivation that resulted in increased IL‐1β secretion and CNS inflammation (Voet *et al*, 2018). CNS‐intrinsic inflammasome activation was further reported in another study that showed caspase‐1‐ and GSDMD‐mediated pyroptosis in microglia, as well as in myelin‐forming oligodendrocytes in the CNS of MS patients and EAE mice (Mckenzie *et al*, 2018).

The relevance of the NLRP3 inflammasome to MS has also been demonstrated in the model of cuprizone‐induced demyelination, in which NLRP3‐deficient mice presented delayed demyelination, oligodendrocyte loss, and neuroinflammation (Jha *et al*, 2010). Additional support was provided by similar observations in caspase‐1 and IL‐18 knockout mice, which unlike IL‐1β knockout mice showed protection (Jha *et al*, 2010). Moreover, IL‐1β and IL‐18 had a differential effect on the process of remyelination after cuprizone treatment, demonstrating a delay in remyelination in IL‐1β knockout mice, whereas accelerated remyelination was seen in IL‐18 knockouts (Jha *et al*, 2010).

## Inflammasome activation in stroke

Neuroinflammation plays a crucial pathological role in stroke, and IL‐1β has been identified as a key cytokine in stroke pathology. So far, four distinct inflammasomes have been implicated in stroke, *viz*. NLRP1, NLRP3, NLRC4, and AIM2 (Barrington *et al*, 2017). Additionally, early studies implicated caspase‐11 in middle cerebral artery occlusion (MCAO), a mouse model of stroke (Kang *et al*, 2000). Expression levels of NLRP3 and NLRP1, as well as cleavage products of caspase‐1, IL‐1β, and IL‐18, were shown to be elevated in postmortem brain tissue of stroke patients (Fann *et al*, 2013). Studies with pharmacological inhibitors of inflammatory caspases and analysis of caspase‐1‐deficient mice—that were later shown to also lack caspase‐11 expression (Kayagaki *et al*, 2011)—showed protection in experimental models of stroke (Friedlander *et al*, 1997; Hara *et al*, 1997; Schielke *et al*, 1998; Rabuffetti *et al*, 2000; Ross *et al*, 2007). In agreement, mice deficient in both IL‐1α and IL‐1β exhibited dramatically reduced ischemic infarct volumes in transient MCAO (Boutin *et al*, 2001), whereas IL‐18‐deficient mice did not show protection in this model (Wheeler *et al*, 2003). NLRP3 deficiency in mice, treatment with the NLRP3 inflammasome inhibitor MCC950/CRID3, and intracerebroventricular injection of NLRP3 siRNAs all ameliorated clinical outcomes in experimental models of stroke (Ma *et al*, 2014; Yang *et al*, 2014; Yuan *et al*, 2015; Ismael *et al*, 2018b). Also, NLRC4‐ and AIM2‐deficient mice were shown to have significantly smaller infarct volumes compared to wild‐type mice subjected to tMCAO, with was associated with strongly reduced microglia cell activation and leukocyte recruitment to the infarct site (Denes *et al*, 2015). Finally, an expression analysis in the peri‐infarct zone of transient MCAO rats revealed elevated levels of the inflammasome components NLRP1, NLRP3, AIM2, NLRC4, and ASC (Lammerding *et al*, 2016), consistent with the potential activation of multiple inflammasomes in stroke.

## Inflammasome activation in traumatic brain and spinal cord injury

Patients with traumatic brain injury (TBI) have significantly higher levels of inflammasome markers in their CSF, including ASC, caspase‐1, NLRP1, and NLRP3 (Adamczak *et al*, 2012; Wallisch *et al*, 2017). However, NLRP1b and ASC knockout mice did not show any improvement in motor recovery or lesion volume in conditions of TBI, compared to control mice, although their levels of IL‐1β were reduced (Brickler *et al*, 2016). NLRP3 expression localizing to neurons, microglia, and astrocytes was also detected in experimental TBI (Liu *et al*, 2013a), and NLRP3‐deficient mice as well as pharmacological blockade of NLRP3 activation were shown to improve recovery from TBI (Irrera *et al*, 2017). Treatment with IL‐1β neutralizing antibodies also was shown to improve the cognitive outcome following TBI (Clausen *et al*, 2009). In contrast to IL‐1β levels, which rapidly increase after TBI, IL‐18 levels gradually increase over several days, suggesting that IL‐1β may be involved in the early phase of TBI, while IL‐18 may contribute to pathogenesis at later phases of the disease. In agreement, IL‐18 inhibition resulted in improved neurologic recovery after 7 days with little effect on the early response being observed immediately after injury (Yatsiv *et al*, 2002).

Similar to TBI, spinal cord injury (SCI) induced higher levels of NLRP1, ASC, caspase‐1, IL‐1β, and IL‐18 in mice, and therapeutic neutralization of ASC was proposed to result in tissue sparing and functional improvement due to reduced inflammasome activation in neurons (de Rivero Vaccari *et al*, 2008). Furthermore, expression of heme oxygenase‐1 was shown to suppress NLRP1 inflammasome activation and neuronal cell death, and lead to improved functional recovery after SCI (Lin *et al*, 2016). Also, enhanced NLRP3 expression can be demonstrated in SCI, predominantly in neurons, but also in microglia and astrocytes (Zendedel *et al*, 2016).

## Inflammasome activation in Alzheimer's disease

Alzheimer's disease (AD) is the most common age‐related neurodegenerative disorder and is characterized by progressive memory deficits and cognitive impairment (Sala Frigerio & De Strooper, 2016). AD pathology is dominantly explained by the amyloid hypothesis, which suggests that the increasing extracellular deposition of misfolded amyloid‐beta (Aβ) is the primary cause of the disease. Although there is overwhelming evidence for a pathogenic role for Aβ in AD, neuroinflammation is increasingly regarded as another key component that actively contributes to AD pathology (De Strooper & Karran, 2016; Sala Frigerio & De Strooper, 2016). Neuroinflammation in AD is primarily driven by the CNS‐resident microglia population (Sarlus & Heneka, 2017; Shi & Holtzman, 2018). Although it is recognized that microglia may exert benign and reparative activities in AD through the phagocytic removal of Aβ deposits, the accumulation of Aβ may also prime microglial cells and promote their activation and production of inflammatory mediators. Moreover, upon Aβ accumulation, microglial cells may become progressively impaired in their ability to phagocytose Aβ (Sarlus & Heneka, 2017; Shi & Holtzman, 2018). Furthermore, a microglial inflammatory state would also impact on other CNS‐resident cells and result in synaptic dysfunction and neuronal damage.

Many recent studies have implicated inflammasomes in the development of AD. Elevated expression of IL‐1β has been reported in microglia that surround Aβ plaques of AD patients (Griffin *et al*, 1989; Simard *et al*, 2006). Additionally, gene expression analysis of cultured PBMCs from AD patients revealed higher expression of NLRP3, ASC, caspase‐1, and caspase‐5 as well as the cytokines IL‐1β and IL‐18 (Saresella *et al*, 2016). The expression levels of NLRC4 and ASC were also found significantly elevated in brain samples of a subgroup of sporadic AD patients (Liu & Chan, 2014). Finally, genetic variants of NLRP1 have been associated with AD risk (Pontillo *et al*, 2012), and the amount of NLRP1 immunopositive neurons was strongly increased in AD brain (Kaushal *et al*, 2015).

*In vitro* studies have shown that fibrillar Aβ activates the NLRP3 inflammasome when phagocytosed by microglia, leading to activation of caspase‐1 and release of IL‐1β (Halle *et al*, 2008). NLRP3 inflammasome activation has also been documented *in vivo* in the transgenic APP/PS1 mouse model of AD, and deficiency in NLRP3 significantly ameliorated spatial memory deficits and hyperactive behavior in these mice, which was associated with reduced hippocampal and cortical Aβ deposition, smaller plaque volumes, decreased levels of pro‐inflammatory cytokines, and improved microglial phagocytic ability (Heneka *et al*, 2013). Similar results were obtained in APP/PS1 mice that were deficient in caspase‐1 (Heneka *et al*, 2013). Inhibition of the NLRP3 inflammasome by MCC950/CRID3 also promoted amyloid‐β clearance and ameliorated cognitive function in APP/PS1 mice (Dempsey *et al*, 2017). Indirect inhibition of NLRP3 inflammasome activation by clinically approved fenamate non‐steroidal anti‐inflammatory drugs that target cyclooxygenase enzymes and volume‐regulated anion channels (VRAC) also was shown to suppress microglia‐mediated neuroinflammation and memory loss in 3×TgAD mice (Daniels *et al*, 2016). Finally, pharmacological inhibition of caspase‐1 activity by VX‐765 reduced amyloid‐β accumulation, brain inflammation, and cognitive impairment (Flores *et al*, 2018). Collectively, these findings suggest that misfolded Aβ activates the microglial NLRP3 inflammasome, which triggers the release of pro‐inflammatory factors that perpetrate a chronic neuroinflammatory environment and promote AD pathology. In addition, NLRP3 inflammasome activity also results in the extracellular release of micrometer‐sized ASC particles that may function as danger signals and alert surrounding macrophages (Baroja‐Mazo *et al*, 2014). These ASC specks were shown to physically bind to Aβ to seed and spread Aβ pathology in a prion‐like manner by promoting misfolded protein aggregation and plaque formation in the APP/PS1 AD model (Venegas *et al*, 2017).

A pathogenic role for IL‐1β in AD has been demonstrated in mice with a deficiency in IL‐1 receptor antagonist (IL‐1ra), which resulted in increased vulnerability to intracerebroventricular injection with human oligomeric Aβ1–42 (Craft *et al*, 2005). Another study showed that IL‐1β injection in the cerebral hemisphere increases Aβ‐APP protein levels in wild‐type mice (Sheng *et al*, 1996). In contrast, sustained hippocampal IL‐1β overexpression in APP/PS1 mice was shown to reduce plaque pathology, an observation that might be explained by the increased phagocytic activity of microglia and macrophages (Shaftel *et al*, 2007). Similar observations were done in 3xTgAD mice, but tau phosphorylation was also increased in these mice, demonstrating the complex role of IL‐1 signaling in AD (Ghosh *et al*, 2013). IL‐1R1 deficiency in mice had no effect on Aβ deposition in Tg2567 AD mice (Das *et al*, 2006). Similarly, IL‐18 deletion did not confer a protective effect in APP/PS1 mice (Tzeng *et al*, 2018). However, these mice were shown to be highly susceptible to grand‐mal seizures and exhibited increased excitatory synaptic proteins and dendritic spine density, an imbalance which leads to a higher basal excitatory synaptic transmission (Tzeng *et al*, 2018). Therefore, IL‐18 may have an unexpected role in controlling neuronal activity.

Although the focus has been on the role of NLRP3 in AD, the role of other inflammasomes has also been characterized in the context of AD. The AIM2 inflammasome was shown to increase Aβ deposition, microglia activation, and cytokine production, but does not affect behavior or memory in transgenic 5xFAD mice (Wu *et al*, 2017). NLRP1 knockdown in APP/PS1 mice was shown to result in significantly reduced neuronal pyroptosis and to rescue cognitive impairments (Tan *et al*, 2014). Finally, the NLRC4 inflammasome was shown to enhance neuronal Aβ levels through activated pro‐inflammatory astrocytes (Liu & Chan, 2014).

## Inflammasome activation in amyotrophic lateral sclerosis

Amyotrophic lateral sclerosis (ALS) is a fatal neurodegenerative disease that is caused by the progressive loss of upper and lower motor neurons and leads to muscle weakness and wasting (Taylor *et al*, 2016). Twenty percent of familial ALS cases are caused by mutations in the gene that encodes superoxide dismutase 1 (SOD1), and many experimental models of ALS rely on expression of mutant SOD1. Deficiency in caspase‐1 and IL‐1β, as well as treatment with the recombinant IL‐1 receptor antagonist anakinra, all significantly extended the lifespan of mutant SOD1 transgenic animals (Meissner *et al*, 2010). Consistently, the expression of NLRP3, NLRC4, AIM2, and caspase‐1 activation has been demonstrated in neural tissue of mutant SOD1 transgenic animals (Pasinelli *et al*, 1998; Johann *et al*, 2015; Gugliandolo *et al*, 2018), and mutant SOD1 was shown to activate caspase‐1 and IL‐1β in microglia (Meissner *et al*, 2010; Lehmann *et al*, 2018). Increased caspase‐1 levels have also been detected in serum of ALS patients (Iłżecka *et al*, 2001), and analysis of postmortem spinal cord tissue showed increased NLRP3, ASC, caspase‐1, and IL‐18 expression levels, with spinal cord astrocytes identified as the main NLRP3 inflammasome‐expressing cell type (Johann *et al*, 2015). However, clinical studies with anakinra in ALS patients have not shown a significant reduction in disease progression (Maier *et al*, 2015), suggesting that inflammasome activation does not play a major role in ALS, or that the pathology is driven by IL‐18 or by DAMPs via pyroptosis.

## Inflammasome activation in Parkinson's disease

Parkinson's disease (PD) is a progressive neurodegenerative disorder caused by the loss of dopaminergic neurons in the substantia nigra pars compacta. Lewy bodies are pathological hallmarks of the disease that predominantly consist of intra‐neuronal aggregates of fibrillar α‐synuclein (Przedborski, 2017). Analysis of the serum of PD patients revealed increased levels of IL‐1β and caspase‐1 (Zhou *et al*, 2016), and elevated IL‐1β levels were observed in the striata of PD patients (Mogi *et al*, 1994). The midbrain of A53T transgenic mice, which model PD based on the overexpression of mutant human A53T α‐synuclein, also contained increased IL‐1β concentrations (Zhou *et al*, 2016). Chronic expression of IL‐1β in the substantia nigra of rats by a recombinant adenovirus expressing IL‐1β was shown to induce progressive death of dopaminergic neurons and resulted in motor impairments (Ferrari *et al*, 2006). However, most of the evidence linking PD to inflammasome signaling comes from *in vitro* studies, and the importance of inflammasomes for the disease is not completely understood. α‐Synuclein was shown to trigger activation of the NLRP3 inflammasome in human monocytes and BV2 microglial cells (Codolo *et al*, 2013; Gustot *et al*, 2015; Zhou *et al*, 2016), but not in primary microglia (Gustin *et al*, 2015). *In vivo*, microinjection of the caspase‐1 inhibitor Ac‐YVAD‐CMK was shown to reduce the expression of NLRP3 inflammasome signaling proteins and improve the number of dopaminergic neurons in LPS‐induced and 6‐hydroxydopamine‐induced PD in rats (Mao *et al*, 2017). In agreement, NLRP3 knockout mice were resistant to the loss of nigral dopaminergic neurons induced by treatment with the neurotoxin 1‐methyl‐4‐phenyl‐1,2,3,6‐tetrahydropyridine (MPTP), which was associated with a reduction in caspase‐1 activation and IL‐1β and IL‐18 secretion (Yan *et al*, 2015). Recently, caspase‐1 was shown to cleave α‐synuclein *in vitro*, generating an aggregation‐prone protein that was toxic to cultured neurons (Wang *et al*, 2016). Finally, mutations in *Parkin, PARK2*,* PARK6,* and *PINK1* have been identified in patients with autosomal recessive early‐onset PD, and microglia and macrophages from PARK2 and PINK1 knockout mice and patients with PARK2 mutations have been shown to display an exacerbated NLRP3 inflammasome response, possibly due to impaired expression of the anti‐inflammatory protein A20 that negatively regulates NLRP3 inflammasome activation (Mouton‐Liger *et al*, 2018).

## Inflammasome activation in prion disease

Prion diseases are a group of rapidly progressive neurodegenerative disorders caused by misfolded aggregated infectious prion proteins (PrP) (Sigurdson *et al*, 2018). IL‐1β levels are increased in the CSF of patients with Creutzfeldt–Jakob disease (CJD) (Van Everbroeck *et al*, 2002), and IL‐1β and cleaved caspase‐1 levels were reported to be upregulated in the brains of scrapie‐infected mice (Schultz *et al*, 2004). Stimulation of microglia with an amyloidogenic PrP peptide induced NLRP3 activation and IL‐1β secretion *in vitro* (Shi *et al*, 2012). Stimulation of microglia with PrP fibrils was also shown to induce toxicity in neurons (Hafner‐Bratkovič *et al*, 2012). Mechanistically, NLRP3 inflammasome activation was suggested to negatively regulate autophagy in microglia, thereby contributing to neurodegeneration (Lai *et al*, 2018). Inhibition of IL‐1R signaling with anakinra was shown to reduce seizure susceptibility in a CJD mouse model (Bertani *et al*, 2017). However, genetic ablation of NLRP3 or ASC in mice did not affect prion pathogenesis, suggesting that the NLRP3 inflammasome does not play a significant role in prion disease (Nuvolone *et al*, 2015).

## Inflammasome activation in Huntington's disease

Huntington's disease (HD) is an autosomal dominant progressive neurodegenerative disease that is caused by the expansion of a trinucleotide CAG repeat in the 5′ coding region of the huntingtin gene, leading to the expression of an abnormal protein that gradually damages cells in the brain (Caron *et al*, 2018). Caspase‐1 activation can be detected in the brains of HD patients and in mouse models of HD, and inhibition of caspase‐1 was shown to slow down disease progression in the R6/2 mouse model of HD (Ona *et al*, 1999). Mechanistically, caspase‐1 was shown to cleave mutant and wild‐type huntingtin *in vitro* (Wellington *et al*, 1998) and *in vivo* (Ona *et al*, 1999), potentially contributing to the neurodegeneration seen in HD. Similarly, treatment with the tetracycline derivative minocycline was shown to delay disease progression by inhibition of caspase‐1 and caspase‐3 expression (Chen *et al*, 2000).

## Pharmacological targeting of inflammasomes

The central role of inflammasomes in neuroinflammatory responses, in particular NLRP3, makes it an attractive drug target for neurodegenerative diseases. Currently approved therapies in the clinic target the downstream effector cytokines released by inflammasome activation, such as anakinra (IL‐1 receptor antagonist), canakinumab (IL‐1β neutralizing antibody), and rilonacept (soluble decoy receptor for IL‐1β and IL‐1α) (Dinarello *et al*, 2012). While valuable in treating autoinflammatory diseases (Van Gorp *et al*, 2019), these IL‐1 inhibitors do not prevent pyroptosis and IL‐18‐driven immune activation. The need for subcutaneous administration of these biological agents represents another drawback of these biological agents (Moran *et al*, 2013; Rossi‐Semerano *et al*, 2015), especially in the case of anakinra, which requires daily injections due to a short half‐life of around 4–6 h (Granowitz *et al*, 1992). Moreover, constitutive neutralization of IL‐1β‐driven signaling increases the risk for infections, as observed in the recent CANTOS trial (Ridker *et al*, 2017). Finally, the concentration of anakinra in CSF of healthy non‐human primates did not surpass 0.2–0.3% of the peripheral serum concentration, demonstrating poor penetration of the BBB (Fox *et al*, 2010). These observations urge for the development of small molecule inflammasome antagonists with improved pharmacokinetic properties and efficacy, and that are able to cross the BBB.

Several small molecule inhibitors that target the inflammasome components caspase‐1, NLRP3, and GSDMD have been reported (Table 2). Since caspase‐1 activation is common to all inflammasome complexes, caspase‐1 inhibition would serve as a pan‐inflammasome inhibitory strategy, while compounds targeting NLRP3 have a narrower selectivity profile. Targeting the inflammasome adaptor ASC would have an intermediate selectivity profile; however, compounds that target ASC have not been reported to date (Mangan *et al*, 2018). A number of NLRP3 inhibitory compounds have been identified and validated in a suite of disease models (Table 2). However, for most agents it is not known whether they directly target NLRP3 or indirectly act on the pathway via other mechanisms, hampering clinical development of these molecules (Mangan *et al*, 2018). To date, only very few compounds targeting NLRP3 or caspase‐1 have entered clinical trials. RP‐1127, an intravenous formulation of the NLRP3 inflammasome inhibitor glyburide (Lamkanfi *et al*, 2009), is being tested in a clinical trial for stroke (EudraCT 2017‐004854‐41) after a positively evaluated pilot study (ClinicalTrials.gov: NCT01268683) (Sheth *et al*, 2014). RP‐1127 is also being tested in a clinical trial for TBI (ClinicalTrials.gov: NCT01454154). In addition, the caspase‐1 inhibitor VX‐765 entered a phase II clinical trial for use in epilepsy (ClinicalTrials.gov: NCT01048255) and psoriasis (ClinicalTrials.gov: NCT00205465), but further development has been discontinued (Mackenzie *et al*, 2010). The encouraging activity of small molecule NLRP3 inflammasome antagonists in preclinical studies warrants the development of inflammasome‐targeting therapies for clinical use.

**Table 2:** Overview of pharmacological compounds targeting inflammasome signaling

Compounds	Benefit (+)/detriment (−)	Neurodegenerative disease model
**Caspase‐1 inhibitors**		
VX‐765 and VX‐740	(−) No further development after phase II clinical trial for use in epilepsy and psoriasis (Mangan *et al*, 2018)	Alzheimer disease (Flores *et al*, 2018) EAE (Mckenzie *et al*, 2018)
**NLRP3 inflammasome inhibitors**		
*Sulfonylurea‐based compounds*		
Glyburide	(+) Specific for NLRP3 inflammasomes; significantly delays LPS‐induced mortality (Lamkanfi *et al*, 2009) (−) High dosage required (Marchetti *et al*, 2014); cardiovascular side effects (Riddle, 2003)	TBI (Simard *et al*, 2012) Ischemic stroke (Simard *et al*, 2012)
CP‐412,245 and CP‐424,174	(+) Oral administration of CP‐424,174 selectively blocks IL‐1 production in mice (Perregaux *et al*, 2001)	
CRID1 and CRID2	(−) No *in vivo* evidence	
MCC950 (also known as CRID3 or CP‐456,773)	(+) NLRP3 inflammasome specific (Coll *et al*, 2015); inhibits NLRP3 activation by all known stimuli (Mangan *et al*, 2018) (−) Precise molecular target unknown	Alzheimer disease (Dempsey *et al*, 2017) EAE (Coll *et al*, 2015) TBI (Ismael *et al*, 2018a; Xu *et al*, 2018) Stroke (Ismael *et al*, 2018b; Ren *et al*, 2018)
16673‐34‐0	(+) Lacks cyclohexylurea group responsible for hypoglycemic activity; prevents NLRP‐mediated myocardial injury (Marchetti *et al*, 2014)	
Hybrid molecules (combining MCC950 and glyburide)	(−) Moderately effective at inhibiting NLRP3 compared to MCC950 (Hill *et al*, 2017)	
*Fenamate classes of NSAIDs*		
Flufenamic and mefenamic acid	(+) Inhibits NLRP3 by blocking VRACs (Daniels *et al*, 2016) (−) Lack of specificity, inhibits multiple inflammatory nodes (Daniels *et al*, 2016)	Alzheimer disease (Daniels *et al*, 2016)
*Michael acceptors*		
Parthenolide	(−) No suitable pharmacological properties (Baldwin *et al*, 2016); inhibits NF‐κB‐dependent signaling (Saadane *et al*, 2007)	Stroke (Dong *et al*, 2013)
BAY 11‐7082	(−) Not specific; inhibits NF‐κB‐dependent signaling (Lee *et al*, 2012)	TBI (Irrera *et al*, 2017)
3,4‐methylenedioxy‐β‐nitrostyrene (MNS)	(−) Modest potency (Baldwin *et al*, 2016)	
Acrylate and acrylamide derivatives (ex. IFN58, IFN39)	(+) Oral administration of IFN39 alleviates DNBS‐induced colitis in rats (Cocco *et al*, 2017)	
*Novel boron compound series*		
NBC13	(+) Significantly decreases LPS‐induced IL‐1β production *in vivo* (Baldwin *et al*, 2017)	
*Other NLRP3 inhibitors*		
Fc11a‐2	(+) *In vivo* efficacy in DSS‐induced colitis (Liu *et al*, 2013b)	
CY‐09	(+) Therapeutic effect in mouse models of CAPS and type 2 diabetes (Jiang *et al*, 2017)	
JC‐171	(+) Treatment effective in EAE in both prophylactic and therapeutic settings (Guo *et al*, 2017)	EAE (Guo *et al*, 2017)
OLT‐177	(+) No adverse effects in preliminary clinical testing of healthy humans (Marchetti *et al*, 2018)	
β‐hydroxybutyrate (BHB)	(−) Not specific for NLRP3; can inhibit HDACs (Youm *et al*, 2015)	
**GSDMD inhibitors**		
Antabuse	(+) Efficacious in sepsis models (preprint: Hu *et al*, 2018)	
Necrosulfonamide	(+) Efficacious in sepsis models (Rathkey *et al*, 2018)	

Pyroptosis is emerging as another key mechanism that contributes to inflammasome‐driven pathology in metabolic, autoimmune, and neurodegenerative diseases. Cleavage of GSDMD by the inflammatory caspase‐1 and caspase‐11 (mouse), or caspase‐1, caspase‐4, and caspase‐5 (human) releases an amino‐terminal pore‐forming GSDMD domain that oligomerizes and inserts in the plasma membrane, inducing membrane rupture and leakage of the cytosolic contents (Vande Walle & Lamkanfi, 2016). Hence, GSDMD inhibition has been proposed as a novel therapeutic strategy to prevent inflammasome‐driven pathology in different diseases. Pyroptosis has recently been shown to be the critical mechanism of IL‐1β‐mediated systemic pathology in the autoinflammatory disease Familial Mediterranean Fever (FMF), which is caused by missense mutations in *Mefv* that activates the pyrin inflammasome (Kanneganti *et al*, 2018). Similarly, GSDMD was shown to control inflammasome‐driven pathology in neonatal‐onset multisystem inflammatory disease (NOMID), which is caused by activating mutations in the inflammasome sensor *NLRP3* (Xiao *et al*, 2018). Indeed, FMF knockin mice and NOMID mice were shown to be fully protected from developing the respective autoinflammatory syndromes when crossed into a Gsdmd‐deficient background (Kanneganti *et al*, 2018; Xiao *et al*, 2018). GSDMD was also demonstrated to play a key role in the pathogenesis of steatohepatitis by controlling cytokine secretion, and Gsdmd knockout mice exhibit decreased severity of steatosis and inflammation in a high‐fat diet‐induced model of non‐alcoholic steatohepatitis (Xu *et al*, 2017). Pharmacologic inhibition of GSDMD by necrosulfonamide also was efficacious in sepsis models (Rathkey *et al*, 2018). Finally, antabuse (disulfiram), a drug used to treat alcohol addiction, was shown to inhibit GSDMD pore formation and IL‐1β secretion in human and mouse cells, and LPS‐induced septic death and IL‐1β secretion in mice (preprint: Hu *et al*, 2018). Together, these observations suggest that inhibition of GSDMD‐dependent pyroptosis may also hold promise for the treatment of inflammasome‐mediated neuroinflammatory pathology.

## Concluding remarks and future perspectives

Although only discovered in 2002 (Martinon *et al*, 2002), inflammasomes are now widely regarded as central regulators of innate immunity playing an indispensable role in the host defense against stress conditions and pathogens that might be harmful to the host. However, inflammasome activation leading to excessive or prolonged inflammation can also contribute to a damaging and destructive environment resulting in the development of inflammatory diseases, including neurodegenerative diseases (Lamkanfi & Dixit, 2012).

Most research in the field has focused on the role of the NLRP3 inflammasome, largely neglecting the potential importance of other inflammasome complexes in CNS disorders. Future research will have to investigate and characterize less well‐known inflammasomes and expand the understanding of inflammasome signaling and its importance for neurodegeneration. The generation of new mouse models and reagents that allow selective targeting of the inflammatory mechanisms initiated by these inflammasomes will be crucial to this end. Also, although the importance of inflammasome activation has been demonstrated in many neurodegenerative diseases and experimental model systems, their specific role in the different CNS cell types is not always clear. Most research has focused on microglia, but other brain cell types also have functional inflammasomes which may contribute to disease pathology (Walsh *et al*, 2014). Since most studies published so far have investigated inflammasome activation using conventional “full body” knockout mice which do not allow to specify the relevant cell types involved, cell type‐specific inflammasome mutant mice will be needed to address the importance of inflammasome signaling in CNS‐specific cell types, assessing their importance for cytokine production, pyroptosis, and CNS inflammation. Also, new techniques including single‐cell RNA sequencing and mass cytometry, in combination with high‐resolution microscopy and *in vivo* cell imaging, should help to get a better view on the spectrum of inflammasomes that is expressed in the different CNS‐resident cell types in different (pathological) conditions.

Major progress needs to be made in translating findings from animal studies to humans. However, key differences exist between human and mouse inflammasomes. Humans have only a single *NLRP1* gene, while mice have three isoforms of *Nlrp1*. Similarly, humans have two orthologues for caspase‐11, *viz*. caspase‐4 and caspase‐5 (Lamkanfi & Dixit, 2012). More research using human cell types, eventually based on iPS technology and patient‐derived material, will be needed to translate findings more effectively from rodents to the human conditions they model.

Finally, since inflammasome signaling is central to many neurodegenerative diseases, targeting their activation is regarded as a potential therapy to treat these diseases. Targeting the downstream IL‐1β and IL‐18 cytokines is probably not the best approach since these cytokines are needed for optimal immunity in the host. Moreover, cytokine‐targeting biologics have limited capacity to penetrate the blood–brain barrier. Therefore, modulating the activity of specific inflammasome sensors and cell type‐specific inflammasome targeting with small molecules will be vital in this context and may have better potential as therapies to treat patients that suffer from neurodegenerative diseases (Guo *et al*, 2015). Also, pyroptosis is now being identified as a critical mechanism driving inflammatory pathology, suggesting GSDMD inhibition as a potential anti‐inflammatory strategy (Kanneganti *et al*, 2018; Mckenzie *et al*, 2018; Xiao *et al*, 2018).

## Conflict of interest

The authors declare that they have no conflict of interest.

**Pending issues**

- The cell type‐specific contribution of inflammasome signaling to neurodegenerative diseases, both peripheral and CNS resident.
- Direct evidence for inflammasome activation and signaling in patients with neurodegenerative disease.
- Characterization of the central activation mechanism and high‐resolution structure of inflammasome proteins.
- Development of compounds able to cross the BBB and that selectively inhibits those inflammasomes that contribute to the pathogenicity.

## References

[emmm201810248-bib-0001] Adamczak S , Dale G , de Rivero Vaccari JP , Bullock MR , Dietrich WD , Keane RW (2012) Inflammasome proteins in cerebrospinal fluid of brain‐injured patients as biomarkers of functional outcome. J Neurosurg 117: 1119–1125 23061392 10.3171/2012.9.JNS12815 PMC3576729

[emmm201810248-bib-0002] Alboni S , Cervia D , Sugama S , Conti B (2010) Interleukin 18 in the CNS. J Neuroinflammation 7: 9 20113500 10.1186/1742-2094-7-9 PMC2830964

[emmm201810248-bib-0003] Allan SM , Tyrrell PJ , Rothwell NJ (2005) Interleukin‐1 and neuronal injury. Nat Rev Immunol 5: 629–640 16034365 10.1038/nri1664

[emmm201810248-bib-0004] Baecher‐Allan C , Kaskow BJ , Weiner HL (2018) Multiple sclerosis: mechanisms and immunotherapy. Neuron 97: 742–768 29470968 10.1016/j.neuron.2018.01.021

[emmm201810248-bib-0005] Baldwin AG , Brough D , Freeman S (2016) Inhibiting the inflammasome: a chemical perspective. J Med Chem 59: 1691–1710 26422006 10.1021/acs.jmedchem.5b01091

[emmm201810248-bib-0006] Baldwin AG , Rivers‐Auty J , Daniels MJD , White CS , Schwalbe CH , Schilling T , Hammadi H , Jaiyong P , Spencer NG , England H *et al* (2017) Boron‐based inhibitors of the NLRP3 inflammasome. Cell Chem Biol 24: 1321–1335.e5 28943355 10.1016/j.chembiol.2017.08.011 PMC5696570

[emmm201810248-bib-0007] Baroja‐Mazo A , Martín‐Sánchez F , Gomez AI , Martínez CM , Amores‐Iniesta J , Compan V , Barberà‐Cremades M , Yagüe J , Ruiz‐Ortiz E , Antón J *et al* (2014) The NLRP3 inflammasome is released as a particulate danger signal that amplifies the inflammatory response. Nat Immunol 15: 738–748 24952504 10.1038/ni.2919

[emmm201810248-bib-0008] Barrington J , Lemarchand E , Allan SM (2017) A brain in flame; do inflammasomes and pyroptosis influence stroke pathology? Brain Pathol 27: 205–212 27997059 10.1111/bpa.12476 PMC8028888

[emmm201810248-bib-0009] Bertani I , Iori V , Trusel M , Maroso M , Foray C , Mantovani S , Tonini R , Vezzani A , Chiesa R (2017) Inhibition of IL‐1β signaling normalizes NMDA‐dependent neurotransmission and reduces seizure susceptibility in a mouse model of Creutzfeldt‐Jakob disease. J Neurosci 37: 10278–10289 28924012 10.1523/JNEUROSCI.1301-17.2017 PMC6596629

[emmm201810248-bib-0010] Boutin H , LeFeuvre RA , Horai R , Asano M , Iwakura Y , Rothwell NJ (2001) Role of IL‐1α and IL‐1β in ischemic brain damage. J Neurosci 21: 5528–5534 11466424 10.1523/JNEUROSCI.21-15-05528.2001 PMC6762680

[emmm201810248-bib-0011] Boyden ED , Dietrich WF (2006) Nalp1b controls mouse macrophage susceptibility to anthrax lethal toxin. Nat Genet 38: 240–244 16429160 10.1038/ng1724

[emmm201810248-bib-0012] Brickler T , Gresham K , Meza A , Coutermarsh‐Ott S , Williams TM , Rothschild DE , Allen IC , Theus MH (2016) Nonessential role for the NLRP1 inflammasome complex in a murine model of traumatic brain injury. Mediators Inflamm 2016: 1–11 10.1155/2016/6373506 PMC4854993 27199506

[emmm201810248-bib-0013] Broz P , von Moltke J , Jones JW , Vance RE , Monack DM (2010) Differential requirement for caspase‐1 autoproteolysis in pathogen‐induced cell death and cytokine processing. Cell Host Microbe 8: 471–483 21147462 10.1016/j.chom.2010.11.007 PMC3016200

[emmm201810248-bib-0014] Broz P , Dixit VM (2016) Inflammasomes: mechanism of assembly, regulation and signalling. Nat Rev Immunol 16: 407–420 27291964 10.1038/nri.2016.58

[emmm201810248-bib-0015] Caron NS , Dorsey ER , Hayden MR (2018) Therapeutic approaches to Huntington disease: from the bench to the clinic. Nat Rev Drug Discov 17: 729–750 30237454 10.1038/nrd.2018.133

[emmm201810248-bib-0017] Chen M , Ona VO , Li M , Ferrante RJ , Fink KB , Zhu S , Bian J , Guo L , Farrell LA , Hersch SM *et al* (2000) Minocycline inhibits caspase‐1 and caspase‐3 expression and delays mortality in a transgenic mouse model of Huntington disease. Nat Med 6: 797–801 10888929 10.1038/77528

[emmm201810248-bib-0018] Clausen F , Hånell A , Björk M , Hillered L , Mir AK , Gram H , Marklund N (2009) Neutralization of interleukin‐1β modifies the inflammatory response and improves histological and cognitive outcome following traumatic brain injury in mice. Eur J Neurosci 30: 385–396 19614750 10.1111/j.1460-9568.2009.06820.x

[emmm201810248-bib-0019] Cocco M , Pellegrini C , Martínez‐Banaclocha H , Giorgis M , Marini E , Costale A , Miglio G , Fornai M , Antonioli L , López‐Castejón G *et al* (2017) Development of an acrylate derivative targeting the NLRP3 inflammasome for the treatment of inflammatory bowel disease. J Med Chem 60: 3656–3671 28410442 10.1021/acs.jmedchem.6b01624

[emmm201810248-bib-0020] Codolo G , Plotegher N , Pozzobon T , Brucale M , Tessari I , Bubacco L , de Bernard M (2013) Triggering of inflammasome by aggregated α‐synuclein, an inflammatory response in synucleinopathies. PLoS ONE 8: e55375 23383169 10.1371/journal.pone.0055375 PMC3561263

[emmm201810248-bib-0021] Coll RC , Robertson AAB , Chae JJ , Higgins SC , Muñoz‐Planillo R , Inserra MC , Vetter I , Dungan LS , Monks BG , Stutz A *et al* (2015) A small‐molecule inhibitor of the NLRP3 inflammasome for the treatment of inflammatory diseases. Nat Med 21: 248–255 25686105 10.1038/nm.3806 PMC4392179

[emmm201810248-bib-0022] Craft JM , Watterson DM , Hirsch E , Van Eldik LJ (2005) Interleukin 1 receptor antagonist knockout mice show enhanced microglial activation and neuronal damage induced by intracerebroventricular infusion of human β‐amyloid. J Neuroinflammation 2: 15 10.1186/1742-2094-2-15 PMC1190207 15967035

[emmm201810248-bib-0023] Daniels MJD , Rivers‐Auty J , Schilling T , Spencer NG , Watremez W , Fasolino V , Booth SJ , White CS , Baldwin AG , Freeman S *et al* (2016) Fenamate NSAIDs inhibit the NLRP3 inflammasome and protect against Alzheimer's disease in rodent models. Nat Commun 7: 12504 27509875 10.1038/ncomms12504 PMC4987536

[emmm201810248-bib-0024] Das P , Smithson LA , Price RW , Holloway VM , Levites Y , Chakrabarty P , Golde TE (2006) Interleukin‐1 receptor 1 knockout has no effect on amyloid deposition in Tg2576 mice and does not alter efficacy following Aβ immunotherapy. J Neuroinflammation 3: 17 16872492 10.1186/1742-2094-3-17 PMC1559596

[emmm201810248-bib-0025] De Strooper B , Karran E (2016) The cellular phase of Alzheimer's disease. Cell 164: 603–615 26871627 10.1016/j.cell.2015.12.056

[emmm201810248-bib-0026] Dempsey C , Rubio Araiz A , Bryson KJ , Finucane O , Larkin C , Mills EL , Robertson AAB , Cooper MA , O'Neill LAJ , Lynch MA (2017) Inhibiting the NLRP3 inflammasome with MCC950 promotes non‐phlogistic clearance of amyloid‐β and cognitive function in APP/PS1 mice. Brain Behav Immun 61: 306–316 28003153 10.1016/j.bbi.2016.12.014

[emmm201810248-bib-0027] Denes A , Coutts G , Lénárt N , Cruickshank SM , Pelegrin P , Skinner J , Rothwell N , Allan SM , Brough D (2015) AIM2 and NLRC4 inflammasomes contribute with ASC to acute brain injury independently of NLRP3. Proc Natl Acad Sci USA 112: 4050–4055 25775556 10.1073/pnas.1419090112 PMC4386342

[emmm201810248-bib-0028] Dinarello CA , Simon A , van der Meer JWM (2012) Treating inflammation by blocking interleukin‐1 in a broad spectrum of diseases. Nat Rev Drug Discov 11: 633–652 22850787 10.1038/nrd3800 PMC3644509

[emmm201810248-bib-0029] Dong L , Qiao H , Zhang X , Zhang X , Wang C , Wang L , Cui L , Zhao J , Xing Y , Li Y *et al* (2013) Parthenolide is neuroprotective in rat experimental stroke model: downregulating NF‐κB, phospho‐p38MAPK, and caspase‐1 and ameliorating BBB permeability. Mediators Inflamm 2013: 370804 23935248 10.1155/2013/370804 PMC3725704

[emmm201810248-bib-0030] Fann DY‐W , Lee S‐Y , Manzanero S , Tang S‐C , Gelderblom M , Chunduri P , Bernreuther C , Glatzel M , Cheng Y‐L , Thundyil J *et al* (2013) Intravenous immunoglobulin suppresses NLRP1 and NLRP3 inflammasome‐mediated neuronal death in ischemic stroke. Cell Death Dis 4: e790 24008734 10.1038/cddis.2013.326 PMC3789184

[emmm201810248-bib-0032] Ferrari CC , Pott Godoy MC , Tarelli R , Chertoff M , Depino AM , Pitossi FJ (2006) Progressive neurodegeneration and motor disabilities induced by chronic expression of IL‐1β in the substantia nigra. Neurobiol Dis 24: 183–193 16901708 10.1016/j.nbd.2006.06.013

[emmm201810248-bib-0033] Flores J , Noël A , Foveau B , Lynham J , Lecrux C , LeBlanc AC (2018) Caspase‐1 inhibition alleviates cognitive impairment and neuropathology in an Alzheimer's disease mouse model. Nat Commun 9: 3916 30254377 10.1038/s41467-018-06449-x PMC6156230

[emmm201810248-bib-0034] Fox E , Jayaprakash N , Pham T‐H , Rowley A , McCully CL , Pucino F , Goldbach‐Mansky R (2010) The serum and cerebrospinal fluid pharmacokinetics of anakinra after intravenous administration to non‐human primates. J Neuroimmunol 223: 138–140 20421138 10.1016/j.jneuroim.2010.03.022 PMC2887614

[emmm201810248-bib-0035] Franchi L , Amer A , Body‐Malapel M , Kanneganti T‐D , Özören N , Jagirdar R , Inohara N , Vandenabeele P , Bertin J , Coyle A *et al* (2006) Cytosolic flagellin requires Ipaf for activation of caspase‐1 and interleukin 1β in salmonella‐infected macrophages. Nat Immunol 7: 576–582 16648852 10.1038/ni1346

[emmm201810248-bib-0036] Freeman L , Guo H , David CN , Brickey WJ , Jha S , Ting JP‐Y (2017) NLR members NLRC4 and NLRP3 mediate sterile inflammasome activation in microglia and astrocytes. J Exp Med 214: 1351–1370 28404595 10.1084/jem.20150237 PMC5413320

[emmm201810248-bib-0037] Friedlander RM , Gagliardini V , Hara H , Fink KB , Li W , MacDonald G , Fishman MC , Greenberg AH , Moskowitz MA , Yuan J (1997) Expression of a dominant negative mutant of interleukin‐1β converting enzyme in transgenic mice prevents neuronal cell death induced by trophic factor withdrawal and ischemic brain injury. J Exp Med 185: 933–940 9120399 10.1084/jem.185.5.933 PMC2196165

[emmm201810248-bib-0038] Furlan R , Martino G , Galbiati F , Poliani PL , Smiroldo S , Bergami A , Desina G , Comi G , Flavell R , Su MS *et al* (1999) Caspase‐1 regulates the inflammatory process leading to autoimmune demyelination. J Immunol 163: 2403–2409 10452974

[emmm201810248-bib-0039] Ghosh S , Wu MD , Shaftel SS , Kyrkanides S , LaFerla FM , Olschowka JA , O'Banion MK (2013) Sustained interleukin‐1 overexpression exacerbates tau pathology despite reduced amyloid burden in an Alzheimer's mouse model. J Neurosci 33: 5053–5064 23486975 10.1523/JNEUROSCI.4361-12.2013 PMC3637949

[emmm201810248-bib-0040] Gong Z , Pan J , Shen Q , Li M , Peng Y (2018) Mitochondrial dysfunction induces NLRP3 inflammasome activation during cerebral ischemia/reperfusion injury. J Neuroinflammation 15: 242 30153825 10.1186/s12974-018-1282-6 PMC6114292

[emmm201810248-bib-0041] Granowitz EV , Porat R , Mier JW , Pribble JP , Stiles DM , Bloedow DC , Catalano MA , Wolff SM , Dinarello CA (1992) Pharmacokinetics, safety and immunomodulatory effects of human recombinant interleukin‐1 receptor antagonist in healthy humans. Cytokine 4: 353–360 1420996 10.1016/1043-4666(92)90078-6

[emmm201810248-bib-0042] Griffin WST , Stanley LC , Ling C , White L , MacLeod V , Perrot LJ , White CL , Araoz C (1989) Brain interleukin 1 and S‐100 immunoreactivity are elevated in Down syndrome and Alzheimer disease. Proc Natl Acad Sci USA 86: 7611–7615 2529544 10.1073/pnas.86.19.7611 PMC298116

[emmm201810248-bib-0043] Gris D , Ye Z , Iocca HA , Wen H , Craven RR , Gris P , Huang M , Schneider M , Miller SD , Ting JP‐Y (2010) NLRP3 plays a critical role in the development of experimental autoimmune encephalomyelitis by mediating Th1 and Th17 responses. J Immunol 185: 974–981 20574004 10.4049/jimmunol.0904145 PMC3593010

[emmm201810248-bib-0044] Guey B , Bodnar M , Manié SN , Tardivel A , Petrilli V (2014) Caspase‐1 autoproteolysis is differentially required for NLRP1b and NLRP3 inflammasome function. Proc Natl Acad Sci USA 111: 17254–17259 25404286 10.1073/pnas.1415756111 PMC4260594

[emmm201810248-bib-0045] Gugliandolo A , Giacoppo S , Bramanti P , Mazzon E (2018) NLRP3 inflammasome activation in a transgenic amyotrophic lateral sclerosis model. Inflammation 41: 93–103 28936769 10.1007/s10753-017-0667-5

[emmm201810248-bib-0046] Guo H , Callaway JB , Ting JP‐Y (2015) Inflammasomes: mechanism of action, role in disease, and therapeutics. Nat Med 21: 677–687 26121197 10.1038/nm.3893 PMC4519035

[emmm201810248-bib-0047] Guo C , Fulp JW , Jiang Y , Li X , Chojnacki JE , Wu J , Wang X‐Y , Zhang S (2017) Development and characterization of a hydroxyl‐sulfonamide analogue, 5‐Chloro‐N‐[2‐(4‐hydroxysulfamoyl‐phenyl)‐ethyl]‐2‐methoxy‐benzamide, as a novel NLRP3 inflammasome inhibitor for potential treatment of multiple sclerosis. ACS Chem Neurosci 8: 2194–2201 28653829 10.1021/acschemneuro.7b00124 PMC5672903

[emmm201810248-bib-0048] Gustin A , Kirchmeyer M , Koncina E , Felten P , Losciuto S , Heurtaux T , Tardivel A , Heuschling P , Dostert C (2015) NLRP3 inflammasome is expressed and functional in mouse brain microglia but not in astrocytes. PLoS ONE 10: e0130624 26091541 10.1371/journal.pone.0130624 PMC4474809

[emmm201810248-bib-0049] Gustot A , Gallea JI , Sarroukh R , Celej MS , Ruysschaert J‐M , Raussens V (2015) Amyloid fibrils are the molecular trigger of inflammation in Parkinson's disease. Biochem J 471: 323–333 26272943 10.1042/BJ20150617

[emmm201810248-bib-0050] Hafner‐Bratkovič I , Benčina M , Fitzgerald KA , Golenbock D , Jerala R (2012) NLRP3 inflammasome activation in macrophage cell lines by prion protein fibrils as the source of IL‐1β and neuronal toxicity. Cell Mol Life Sci 69: 4215–4228 22926439 10.1007/s00018-012-1140-0 PMC3508391

[emmm201810248-bib-0051] Halle A , Hornung V , Petzold GC , Stewart CR , Monks BG , Reinheckel T , Fitzgerald K , Latz E , Moore K , Golenbock D (2008) The NALP3 inflammasome is involved in the innate immune response to amyloid‐β. Nat Immunol 9: 857–865 18604209 10.1038/ni.1636 PMC3101478

[emmm201810248-bib-0052] Hara H , Friedlander RM , Gagliardini V , Ayata C , Fink K , Huang Z , Shimizu‐Sasamata M , Yuan J , Moskowitz MA (1997) Inhibition of interleukin 1β converting enzyme family proteases reduces ischemic and excitotoxic neuronal damage. Proc Natl Acad Sci USA 94: 2007–2012 9050895 10.1073/pnas.94.5.2007 PMC20033

[emmm201810248-bib-0053] Heneka MT , Kummer MP , Stutz A , Delekate A , Schwartz S , Vieira‐Saecker A , Griep A , Axt D , Remus A , Tzeng T‐C *et al* (2013) NLRP3 is activated in Alzheimer's disease and contributes to pathology in APP/PS1 mice. Nature 493: 674–678 23254930 10.1038/nature11729 PMC3812809

[emmm201810248-bib-0054] Heneka MT , Kummer MP , Latz E (2014) Innate immune activation in neurodegenerative disease. Nat Rev Immunol 14: 463–477 24962261 10.1038/nri3705

[emmm201810248-bib-0055] Heneka MT , McManus RM , Latz E (2018) Inflammasome signalling in brain function and neurodegenerative disease. Nat Rev Neurosci 19: 610–621 30206330 10.1038/s41583-018-0055-7

[emmm201810248-bib-0056] Hill JR , Coll RC , Sue N , Reid JC , Dou J , Holley CL , Pelingon R , Dickinson JB , Biden TJ , Schroder K *et al* (2017) Sulfonylureas as concomitant insulin secretagogues and NLRP3 inflammasome inhibitors. ChemMedChem 12: 1449–1457 28703484 10.1002/cmdc.201700270

[emmm201810248-bib-0057] Hornung V , Ablasser A , Charrel‐Dennis M , Bauernfeind F , Horvath G , Caffrey DR , Latz E , Fitzgerald KA (2009) AIM2 recognizes cytosolic dsDNA and forms a caspase‐1‐activating inflammasome with ASC. Nature 458: 514–518 19158675 10.1038/nature07725 PMC2726264

[emmm201810248-bib-0600] Hu JJ , Liu X , Zhao J , Xia S , Ruan J , Luo X , Kim J , Lieberman J , Wu H (2018) Identification of pyroptosis inhibitors that target a reactive cysteine in gasdermin D. *bioRxiv* 10.1101/365908 [PREPRINT]

[emmm201810248-bib-0058] Iłżecka J , Stelmasiak Z , Dobosz B (2001) Interleukin‐1β converting enzyme/Caspase‐1 (ICE/Caspase‐1) and soluble APO‐1/Fas/CD95 receptor in amyotrophic lateral sclerosis patients. Acta Neurol Scand 103: 255–258 11328198

[emmm201810248-bib-0059] Inoue M , Williams KL , Gunn MD , Shinohara ML (2012a) NLRP3 inflammasome induces chemotactic immune cell migration to the CNS in experimental autoimmune encephalomyelitis. Proc Natl Acad Sci USA 109: 10480–10485 22699511 10.1073/pnas.1201836109 PMC3387125

[emmm201810248-bib-0060] Inoue M , Williams KL , Oliver T , Vandenabeele P , Rajan J V , Miao EA , Shinohara ML (2012b) Interferon‐β therapy against EAE is effective only when development of the disease depends on the NLRP3 inflammasome. Sci Signal 5: ra38 22623753 10.1126/scisignal.2002767 PMC3509177

[emmm201810248-bib-0061] Inoue M , Shinohara ML (2013) NLRP3 inflammasome and MS/EAE. Autoimmune Dis 2013: 859145 23365725 10.1155/2013/859145 PMC3556409

[emmm201810248-bib-0062] Inoue M , Chen P‐H , Siecinski S , Li Q‐J , Liu C , Steinman L , Gregory SG , Benner E , Shinohara ML (2016) An interferon‐β‐resistant and NLRP3 inflammasome‐independent subtype of EAE with neuronal damage. Nat Neurosci 19: 1599–1609 27820602 10.1038/nn.4421 PMC5482232

[emmm201810248-bib-0063] Irrera N , Pizzino G , Calò M , Pallio G , Mannino F , Famà F , Arcoraci V , Fodale V , David A , Francesca C *et al* (2017) Lack of the Nlrp3 inflammasome improves mice recovery following traumatic brain injury. Front Pharmacol 8: 459 28769794 10.3389/fphar.2017.00459 PMC5509758

[emmm201810248-bib-0064] Ismael S , Nasoohi S , Ishrat T (2018a) MCC950, the selective inhibitor of nucleotide oligomerization domain‐like receptor protein‐3 inflammasome, protects mice against traumatic brain injury. J Neurotrauma 35: 1294–1303 29295651 10.1089/neu.2017.5344 PMC5962912

[emmm201810248-bib-0065] Ismael S , Zhao L , Nasoohi S , Ishrat T (2018b) Inhibition of the NLRP3‐inflammasome as a potential approach for neuroprotection after stroke. Sci Rep 8: 5971 29654318 10.1038/s41598-018-24350-x PMC5899150

[emmm201810248-bib-0066] Jha S , Srivastava SY , Brickey WJ , Iocca H , Toews A , Morrison JP , Chen VS , Gris D , Matsushima GK , Ting JP‐Y (2010) The inflammasome sensor, NLRP3, regulates CNS inflammation and demyelination via caspase‐1 and interleukin‐18. J Neurosci 30: 15811–15820 21106820 10.1523/JNEUROSCI.4088-10.2010 PMC6633756

[emmm201810248-bib-0067] Jiang H , He H , Chen Y , Huang W , Cheng J , Ye J , Wang A , Tao J , Wang C , Liu Q *et al* (2017) Identification of a selective and direct NLRP3 inhibitor to treat inflammatory disorders. J Exp Med 214: 3219–3238 29021150 10.1084/jem.20171419 PMC5679172

[emmm201810248-bib-0068] Johann S , Heitzer M , Kanagaratnam M , Goswami A , Rizo T , Weis J , Troost D , Beyer C (2015) NLRP3 inflammasome is expressed by astrocytes in the SOD1 mouse model of ALS and in human sporadic ALS patients. Glia 63: 2260–2273 26200799 10.1002/glia.22891

[emmm201810248-bib-0069] Kang S‐J , Wang S , Hara H , Peterson EP , Namura S , Amin‐Hanjani S , Huang Z , Srinivasan A , Tomaselli KJ , Thornberry NA *et al* (2000) Dual role of caspase‐11 in mediating activation of caspase‐1 and caspase‐3 under pathological conditions. J Cell Biol 149: 613–622 10791975 10.1083/jcb.149.3.613 PMC2174843

[emmm201810248-bib-0070] Kanneganti A , Malireddi RKS , Saavedra PHV , Vande Walle L , Van Gorp H , Kambara H , Tillman H , Vogel P , Luo HR , Xavier RJ *et al* (2018) GSDMD is critical for autoinflammatory pathology in a mouse model of familial mediterranean fever. J Exp Med 215: 1519–1529 29793924 10.1084/jem.20172060 PMC5987922

[emmm201810248-bib-0071] Kaushal V , Dye R , Pakavathkumar P , Foveau B , Flores J , Hyman B , Ghetti B , Koller BH , LeBlanc AC (2015) Neuronal NLRP1 inflammasome activation of Caspase‐1 coordinately regulates inflammatory interleukin‐1‐beta production and axonal degeneration‐associated Caspase‐6 activation. Cell Death Differ 22: 1676–1686 25744023 10.1038/cdd.2015.16 PMC4563782

[emmm201810248-bib-0072] Kawana N , Yamamoto Y , Ishida T , Saito Y , Konno H , Arima K , Satoh JI (2013) Reactive astrocytes and perivascular macrophages express NLRP3 inflammasome in active demyelinating lesions of multiple sclerosis and necrotic lesions of neuromyelitis optica and cerebral infarction. Clin Exp Neuroimmunol 4: 296–304

[emmm201810248-bib-0073] Kayagaki N , Warming S , Lamkanfi M , Vande Walle L , Louie S , Dong J , Newton K , Qu Y , Liu J , Heldens S *et al* (2011) Non‐canonical inflammasome activation targets caspase‐11. Nature 479: 117–121 22002608 10.1038/nature10558

[emmm201810248-bib-0074] Keane RW , Dietrich WD , de Rivero Vaccari JP (2018) Inflammasome proteins as biomarkers of multiple sclerosis. Front Neurol 9: 135. 29615953 10.3389/fneur.2018.00135 PMC5868457

[emmm201810248-bib-0075] Lai M , Yao H , Shah SZA , Wu W , Wang D , Zhao Y , Wang L , Zhou X , Zhao D , Yang L (2018) The NLRP3‐Caspase 1 inflammasome negatively regulates autophagy via TLR4‐TRIF in prion peptide‐infected microglia. Front Aging Neurosci 10: 116 29720937 10.3389/fnagi.2018.00116 PMC5915529

[emmm201810248-bib-0076] Lamkanfi M , Mueller JL , Vitari AC , Misaghi S , Fedorova A , Deshayes K , Lee WP , Hoffman HM , Dixit VM (2009) Glyburide inhibits the Cryopyrin/Nalp3 inflammasome. J Cell Biol 187: 61–70 19805629 10.1083/jcb.200903124 PMC2762099

[emmm201810248-bib-0077] Lamkanfi M , Dixit VM (2012) Inflammasomes and their roles in health and disease. Annu Rev Cell Dev Biol 28: 137–161 22974247 10.1146/annurev-cellbio-101011-155745

[emmm201810248-bib-0078] Lamkanfi M , Dixit VM (2014) Mechanisms and functions of inflammasomes. Cell 157: 1013–1022 24855941 10.1016/j.cell.2014.04.007

[emmm201810248-bib-0079] Lammerding L , Slowik A , Johann S , Beyer C , Zendedel A (2016) Poststroke inflammasome expression and regulation in the peri‐infarct area by gonadal steroids after transient focal ischemia in the rat brain. Neuroendocrinology 103: 460–475 26337121 10.1159/000439435

[emmm201810248-bib-0080] Lampron A , ElAli A , Rivest S (2013) Innate immunity in the CNS: redefining the relationship between the CNS and its environment. Neuron 78: 214–232 23622060 10.1016/j.neuron.2013.04.005

[emmm201810248-bib-0081] Lee J , Rhee MH , Kim E , Cho JY (2012) BAY 11‐7082 is a broad‐spectrum inhibitor with anti‐inflammatory activity against multiple targets. Mediators Inflamm 2012: 416036 22745523 10.1155/2012/416036 PMC3382285

[emmm201810248-bib-0082] Lehmann S , Esch E , Hartmann P , Goswami A , Nikolin S , Weis J , Beyer C , Johann S (2018) Expression profile of pattern recognition receptors in skeletal muscle of SOD1(G93A) amyotrophic lateral sclerosis (ALS) mice and sporadic ALS patients. Neuropathol Appl Neurobiol 44: 606–627 29575052 10.1111/nan.12483

[emmm201810248-bib-0083] Lévesque SA , Paré A , Mailhot B , Bellver‐Landete V , Kébir H , Lécuyer M‐A , Alvarez JI , Prat A , de Rivero Vaccari JP , Keane RW *et al* (2016) Myeloid cell transmigration across the CNS vasculature triggers IL‐1β–driven neuroinflammation during autoimmune encephalomyelitis in mice. J Exp Med 213: 929–949 27139491 10.1084/jem.20151437 PMC4886360

[emmm201810248-bib-0084] Lin W‐P , Xiong G‐P , Lin Q , Chen X‐W , Zhang L‐Q , Shi J‐X , Ke Q‐F , Lin J‐H (2016) Heme oxygenase‐1 promotes neuron survival through down‐regulation of neuronal NLRP1 expression after spinal cord injury. J Neuroinflammation 13: 52 26925775 10.1186/s12974-016-0521-y PMC4772494

[emmm201810248-bib-0085] Liu H‐D , Li W , Chen Z‐R , Hu Y‐C , Zhang D‐D , Shen W , Zhou M‐L , Zhu L , Hang C‐H (2013a) Expression of the NLRP3 inflammasome in cerebral cortex after traumatic brain injury in a rat model. Neurochem Res 38: 2072–2083 23892989 10.1007/s11064-013-1115-z

[emmm201810248-bib-0086] Liu W , Guo W , Wu J , Luo Q , Tao F , Gu Y , Shen Y , Li J , Tan R , Xu Q *et al* (2013b) A novel benzo[d]imidazole derivate prevents the development of dextran sulfate sodium‐induced murine experimental colitis via inhibition of NLRP3 inflammasome. Biochem Pharmacol 85: 1504–1512 23506741 10.1016/j.bcp.2013.03.008

[emmm201810248-bib-0087] Liu L , Chan C (2014) IPAF inflammasome is involved in interleukin‐1β production from astrocytes, induced by palmitate; implications for Alzheimer's disease. Neurobiol Aging 35: 309–321 24054992 10.1016/j.neurobiolaging.2013.08.016 PMC3832124

[emmm201810248-bib-0088] Ma Q , Chen S , Hu Q , Feng H , Zhang JH , Tang J (2014) NLRP3 inflammasome contributes to inflammation after intracerebral hemorrhage. Ann Neurol 75: 209–219 24273204 10.1002/ana.24070 PMC4386653

[emmm201810248-bib-0089] Mackenzie SH , Schipper JL , Clark AC (2010) The potential for caspases in drug discovery. Curr Opin Drug Discov Devel 13: 568–576 PMC3289102 20812148

[emmm201810248-bib-0090] Maier A , Deigendesch N , Müller K , Weishaupt JH , Krannich A , Röhle R , Meissner F , Molawi K , Münch C , Holm T *et al* (2015) Interleukin‐1 antagonist anakinra in amyotrophic lateral sclerosis‐A pilot study. PLoS ONE 10: e0139684 26444282 10.1371/journal.pone.0139684 PMC4596620

[emmm201810248-bib-0091] Mamik MK , Power C (2017) Inflammasomes in neurological diseases: emerging pathogenic and therapeutic concepts. Brain 140: 2273–2285 29050380 10.1093/brain/awx133

[emmm201810248-bib-0092] Mangan MSJ , Olhava EJ , Roush WR , Seidel HM , Glick GD , Latz E (2018) Targeting the NLRP3 inflammasome in inflammatory diseases. Nat Rev Drug Discov 17: 588–606 30026524 10.1038/nrd.2018.97

[emmm201810248-bib-0093] Mao Z , Liu C , Ji S , Yang Q , Ye H , Han H , Xue Z (2017) The NLRP3 inflammasome is involved in the pathogenesis of Parkinson's disease in rats. Neurochem Res 42: 1104–1115 28247334 10.1007/s11064-017-2185-0

[emmm201810248-bib-0094] Marchetti C , Chojnacki J , Toldo S , Mezzaroma E , Tranchida N , Rose SW , Federici M , Van Tassell BW , Zhang S , Abbate A (2014) A novel pharmacologic inhibitor of the NLRP3 inflammasome limits myocardial injury following ischemia‐reperfusion in the mouse. J Cardiovasc Pharmacol 63: 316–322 24336017 10.1097/FJC.0000000000000053 PMC3980088

[emmm201810248-bib-0095] Marchetti C , Swartzwelter B , Gamboni F , Neff CP , Richter K , Azam T , Carta S , Tengesdal I , Nemkov T , D'Alessandro A *et al* (2018) OLT1177, a β‐sulfonyl nitrile compound, safe in humans, inhibits the NLRP3 inflammasome and reverses the metabolic cost of inflammation. Proc Natl Acad Sci USA 115: E1530–E1539 29378952 10.1073/pnas.1716095115 PMC5816172

[emmm201810248-bib-0096] Martinon F , Burns K , Tschopp J (2002) The inflammasome: a molecular platform triggering activation of inflammatory caspases and processing of proIL‐β. Mol Cell 10: 417–426 12191486 10.1016/s1097-2765(02)00599-3

[emmm201810248-bib-0097] Mckenzie BA , Mamik MK , Saito LB , Boghozian R , Monaco MC , Major EO , Lu J‐Q , Branton WG , Power C (2018) Caspase‐1 inhibition prevents glial inflammasome activation and pyroptosis in models of multiple sclerosis. Proc Natl Acad Sci USA 115: E6065–E6074 29895691 10.1073/pnas.1722041115 PMC6042136

[emmm201810248-bib-0098] Meissner F , Molawi K , Zychlinsky A (2010) Mutant superoxide dismutase 1‐induced IL‐1β accelerates ALS pathogenesis. Proc Natl Acad Sci USA 107: 10346–13050 10.1073/pnas.1002396107 PMC2919927 20616033

[emmm201810248-bib-0099] Miao EA , Alpuche‐Aranda CM , Dors M , Clark AE , Bader MW , Miller SI , Aderem A (2006) Cytoplasmic flagellin activates caspase‐1 and secretion of interleukin 1β via Ipaf. Nat Immunol 7: 569–575 16648853 10.1038/ni1344

[emmm201810248-bib-0100] Miao EA , Mao DP , Yudkovsky N , Bonneau R , Lorang CG , Warren SE , Leaf IA , Aderem A (2010) Innate immune detection of the type III secretion apparatus through the NLRC4 inflammasome. Proc Natl Acad Sci USA 107: 3076–3080 20133635 10.1073/pnas.0913087107 PMC2840275

[emmm201810248-bib-0101] Mogi M , Harada M , Kondo T , Riederer P , Inagaki H , Minami M , Nagatsu T (1994) Interleukin‐1β, interleukin‐6, epidermal growth factor and transforming growth factor‐α are elevated in the brain from parkinsonian patients. Neurosci Lett 180: 147–150 7700568 10.1016/0304-3940(94)90508-8

[emmm201810248-bib-0102] Moran A , Bundy B , Becker DJ , Dimeglio LA , Gitelman SE , Goland R , Greenbaum CJ , Herold KC , Marks JB , Raskin P *et al* (2013) Interleukin‐1 antagonism in type 1 diabetes of recent onset: two multicentre, randomised, double‐blind, placebo‐controlled trials. Lancet 381: 1905–1915 23562090 10.1016/S0140-6736(13)60023-9 PMC3827771

[emmm201810248-bib-0103] Mouton‐Liger F , Rosazza T , Sepulveda‐Diaz J , Ieang A , Hassoun S‐M , Claire E , Mangone G , Brice A , Michel PP , Corvol J‐C *et al* (2018) Parkin deficiency modulates NLRP3 inflammasome activation by attenuating an A20‐dependent negative feedback loop. Glia 66: 1736–1751 29665074 10.1002/glia.23337 PMC6190839

[emmm201810248-bib-0104] Nuvolone M , Sorce S , Schwarz P , Aguzzi A (2015) Prion pathogenesis in the absence of NLRP3/ASC inflammasomes. PLoS ONE 10: e0117208 25671600 10.1371/journal.pone.0117208 PMC4324825

[emmm201810248-bib-0105] Ona VO , Li M , Vonsattel JP , Andrews LJ , Khan SQ , Chung WM , Frey AS , Menon AS , Li X‐J , Stieg PE *et al* (1999) Inhibition of caspase‐1 slows disease progression in a mouse model of Huntington's disease. Nature 399: 263–267 10353249 10.1038/20446

[emmm201810248-bib-0106] Pasinelli P , Borchelt DR , Houseweart MK , Cleveland DW , Brown RHJ (1998) Caspase‐1 is activated in neural cells and tissue with amyotrophic lateral sclerosis‐associated mutations in copper‐zinc superoxide dismutase. Proc Natl Acad Sci USA 95: 15763–15768 9861044 10.1073/pnas.95.26.15763 PMC28118

[emmm201810248-bib-0107] Perregaux DG , Mcniff P , Laliberte R , Hawryluk N , Peurano H , Stam E , Eggler J , Griffiths R , Dombroski MA , Gabel CA (2001) Identification and characterization of a novel class of interleukin‐1 post‐translational processing inhibitors. J Pharmacol Exp Ther 299: 187–197 11561079

[emmm201810248-bib-0108] Pontillo A , Catamo E , Arosio B , Mari D , Crovella S (2012) NALP1/NLRP1 genetic variants are associated with Alzheimer disease. Alzheimer Dis Assoc Disord 26: 277–281 21946017 10.1097/WAD.0b013e318231a8ac

[emmm201810248-bib-0109] Przedborski S (2017) The two‐century journey of Parkinson disease research. Nat Rev Neurosci 18: 251–259 28303016 10.1038/nrn.2017.25

[emmm201810248-bib-0110] Rabuffetti M , Sciorati C , Tarozzo G , Clementi E , Manfredi AA , Beltramo M (2000) Inhibition of caspase‐1‐like activity by Ac‐Tyr‐Val‐Ala‐Asp‐chloromethyl ketone induces long‐lasting neuroprotection in cerebral ischemia through apoptosis reduction and decrease of proinflammatory cytokines. J Neurosci 20: 4398–4404 10844008 10.1523/JNEUROSCI.20-12-04398.2000 PMC6772438

[emmm201810248-bib-0111] Ransohoff RM (2012) Animal models of multiple sclerosis: the good, the bad and the bottom line. Nat Neurosci 15: 1074–1077 22837037 10.1038/nn.3168 PMC7097342

[emmm201810248-bib-0112] Rathkey JK , Zhao J , Liu Z , Chen Y , Yang J , Kondolf HC , Benson BL , Chirieleison SM , Huang AY , Dubyak GR *et al* (2018) Chemical disruption of the pyroptotic pore‐forming protein gasdermin D inhibits inflammatory cell death and sepsis. Sci Immunol 3: eaat2738 30143556 10.1126/sciimmunol.aat2738 PMC6462819

[emmm201810248-bib-0113] Ren H , Kong Y , Liu Z , Zang D , Yang X , Wood K , Li M , Liu Q (2018) Selective NLRP3 (pyrin domain‐containing protein 3) inflammasome inhibitor reduces brain injury after intracerebral hemorrhage. Stroke 49: 184–192 29212744 10.1161/STROKEAHA.117.018904 PMC5753818

[emmm201810248-bib-0114] Riddle M (2003) Editorial: sulfonylureas differ in effects on ischemic preconditioning‐is it time to retire glyburide? J Clin Endocrinol Metab 88: 528–530 12574174 10.1210/jc.2002-021971

[emmm201810248-bib-0115] Ridker PM , Everett BM , Thuren T , MacFadyen JG , Chang WH , Ballantyne C , Fonseca F , Nicolau J , Koenig W , Anker SD *et al* (2017) Antiinflammatory therapy with canakinumab for atherosclerotic disease. N Engl J Med 377: 1119–1131 28845751 10.1056/NEJMoa1707914

[emmm201810248-bib-0116] de Rivero Vaccari JP , Lotocki G , Marcillo AE , Dietrich WD , Keane RW (2008) A molecular platform in neurons regulates inflammation after spinal cord injury. J Neurosci 28: 3404–3414 18367607 10.1523/JNEUROSCI.0157-08.2008 PMC6670583

[emmm201810248-bib-0117] Ross J , Brough D , Gibson RM , Loddick SA , Rothwell NJ (2007) A selective, non‐peptide caspase‐1 inhibitor, VRT‐018858, markedly reduces brain damage induced by transient ischemia in the rat. Neuropharmacology 53: 638–642 17845807 10.1016/j.neuropharm.2007.07.015

[emmm201810248-bib-0118] Rossi‐Semerano L , Fautrel B , Wendling D , Hachulla E , Galeotti C , Semerano L , Touitou I , Koné‐Paut I & Inflammation) M (Maladies A et A‐I‐1) study G on the behalf of C Club R et (2015) Tolerance and efficacy of off‐label anti‐interleukin‐1 treatments in France: a nationwide survey. Orphanet J Rare Dis 10: 19 25758134 10.1186/s13023-015-0228-7 PMC4340831

[emmm201810248-bib-0119] Saadane A , Masters S , Didonato J , Li J , Berger M (2007) Parthenolide inhibits IκB kinase, NF‐κB activation, and inflammatory response in cystic fibrosis cells and mice. Am J Respir Cell Mol Biol 36: 728–736 17272824 10.1165/rcmb.2006-0323OC PMC1899341

[emmm201810248-bib-0120] Sala Frigerio C , De Strooper B (2016) Alzheimer's disease mechanisms and emerging roads to novel therapeutics. Annu Rev Neurosci 39: 57–79 27050320 10.1146/annurev-neuro-070815-014015

[emmm201810248-bib-0121] Saresella M , La Rosa F , Piancone F , Zoppis M , Marventano I , Calabrese E , Rainone V , Nemni R , Mancuso R , Clerici M (2016) The NLRP3 and NLRP1 inflammasomes are activated in Alzheimer's disease. Mol Neurodegener 11: 23 26939933 10.1186/s13024-016-0088-1 PMC4778358

[emmm201810248-bib-0122] Sarlus H , Heneka MT (2017) Microglia in Alzheimer's disease. J Clin Invest 127: 3240–3249 28862638 10.1172/JCI90606 PMC5669553

[emmm201810248-bib-0123] Schielke GP , Yang GY , Shivers BD , Betz AL (1998) Reduced ischemic brain injury in interleukin‐1β converting enzyme‐deficient mice. J Cereb Blood Flow Metab 18: 180–185 9469161 10.1097/00004647-199802000-00009

[emmm201810248-bib-0124] Schultz J , Schwarz A , Neidhold S , Burwinkel M , Riemer C , Simon D , Kopf M , Otto M , Baier M (2004) Role of interleukin‐1 in prion disease‐associated astrocyte activation. Am J Pathol 165: 671–678 15277240 10.1016/S0002-9440(10)63331-7 PMC1618583

[emmm201810248-bib-0125] Shaftel SS , Kyrkanides S , Olschowka JA , Miller JH , Johnson RE , O'Banion MK (2007) Sustained hippocampal IL‐1β overexpression mediates chronic neuroinflammation and ameliorates Alzheimer plaque pathology. J Clin Invest 117: 1595–1604 17549256 10.1172/JCI31450 PMC1878531

[emmm201810248-bib-0126] Sheng JG , Ito K , Skinner RD , Mrak RE , Rovnaghi CR , Van Eldik LJ , Griffin WST (1996) *In vivo* and *in vitro* evidence supporting a role for the inflammatory cytokine interleukin‐1 as a driving force in Alzheimer pathogenesis. Neurobiol Aging 17: 761–766 8892349 10.1016/0197-4580(96)00104-2 PMC3886636

[emmm201810248-bib-0127] Sheth KN , Kimberly WT , Elm JJ , Kent TA , Mandava P , Yoo AJ , Thomalla G , Campbell B , Donnan GA , Davis SM *et al* (2014) Pilot study of intravenous glyburide in patients with a large ischemic stroke. Stroke 45: 281–283 24193798 10.1161/STROKEAHA.113.003352 PMC4235339

[emmm201810248-bib-0128] Shi F , Yang L , Kouadir M , Yang Y , Wang J , Zhou X , Yin X , Zhao D (2012) The NALP3 inflammasome is involved in neurotoxic prion peptide‐induced microglial activation. J Neuroinflammation 9: 73 22531291 10.1186/1742-2094-9-73 PMC3394218

[emmm201810248-bib-0129] Shi J , Gao W , Shao F (2017) Pyroptosis: gasdermin‐mediated programmed necrotic cell death. Trends Biochem Sci 42: 245–254 27932073 10.1016/j.tibs.2016.10.004

[emmm201810248-bib-0130] Shi Y , Holtzman DM (2018) Interplay between innate immunity and Alzheimer disease: APOE and TREM2 in the spotlight. Nat Rev Immunol 18: 759–772 30140051 10.1038/s41577-018-0051-1 PMC6425488

[emmm201810248-bib-0131] Sigurdson CJ , Bartz JC , Glatzel M (2018) Cellular and molecular mechanisms of prion disease. Annu Rev Pathol 14: 497–516 30355150 10.1146/annurev-pathmechdis-012418-013109 PMC9071098

[emmm201810248-bib-0132] Simard AR , Soulet D , Gowing G , Julien J‐P , Rivest S (2006) Bone marrow‐derived microglia play a critical role in restricting senile plaque formation in Alzheimer's disease. Neuron 49: 489–502 16476660 10.1016/j.neuron.2006.01.022

[emmm201810248-bib-0133] Simard JM , Woo SK , Schwartzbauer GT , Gerzanich V (2012) Sulfonylurea receptor 1 in central nervous system injury: a focused review. J Cereb Blood Flow Metab 32: 1699–1717 22714048 10.1038/jcbfm.2012.91 PMC3434627

[emmm201810248-bib-0134] Sutton C , Brereton C , Keogh B , Mills KHG , Lavelle EC (2006) A crucial role for interleukin (IL)‐1 in the induction of IL‐17–producing T cells that mediate autoimmune encephalomyelitis. J Exp Med 203: 1685–1691 16818675 10.1084/jem.20060285 PMC2118338

[emmm201810248-bib-0135] Tan M‐S , Tan L , Jiang T , Zhu X‐C , Wang H‐F , Jia C‐D , Yu J‐T (2014) Amyloid‐β induces NLRP1‐dependent neuronal pyroptosis in models of Alzheimer's disease. Cell Death Dis 5: e1382 25144717 10.1038/cddis.2014.348 PMC4454321

[emmm201810248-bib-0136] Taylor JP , Brown RHJ , Cleveland DW (2016) Decoding ALS: from genes to mechanism. Nature 539: 197–206 27830784 10.1038/nature20413 PMC5585017

[emmm201810248-bib-0137] Tsai S‐J (2017) Effects of interleukin‐1beta polymorphisms on brain function and behavior in healthy and psychiatric disease conditions. Cytokine Growth Factor Rev 37: 89–97 28599834 10.1016/j.cytogfr.2017.06.001

[emmm201810248-bib-0138] Tzeng T‐C , Hasegawa Y , Iguchi R , Cheung A , Caffrey DR , Thatcher EJ , Mao W , Germain G , Tamburro ND , Okabe S *et al* (2018) Inflammasome‐derived cytokine IL18 suppresses amyloid‐induced seizures in Alzheimer‐prone mice. Proc Natl Acad Sci USA 115: 9002–9007 30127003 10.1073/pnas.1801802115 PMC6130368

[emmm201810248-bib-0139] Van Everbroeck B , Dewulf E , Pals P , Lübke U , Martin JJ , Cras P (2002) The role of cytokines, astrocytes, microglia and apoptosis in Creutzfeldt‐Jakob disease. Neurobiol Aging 23: 59–64 11755020 10.1016/s0197-4580(01)00236-6

[emmm201810248-bib-0140] Van Gorp H , Van Opdenbosch N , Lamkanfi M (2019) Inflammasome‐dependent cytokines at the crossroads of health and autoinflammatory disease. Cold Spring Harb Perspect Biol 11: pii: a028563 10.1101/cshperspect.a028563 PMC6314066 29038114

[emmm201810248-bib-0141] Van Opdenbosch N , Gurung P , Vande Walle L , Fossoul A , Kanneganti T‐D , Lamkanfi M (2014) Activation of the NLRP1b inflammasome independently of ASC‐mediated caspase‐1 autoproteolysis and speck formation. Nat Commun 5: 1–14 10.1038/ncomms4209 PMC3926011 24492532

[emmm201810248-bib-0142] Vande Walle L , Van Opdenbosch N , Jacques P , Fossoul A , Verheugen E , Vogel P , Beyaert R , Elewaut D , Kanneganti T‐D , van Loo G *et al* (2014) Negative regulation of the NLRP3 inflammasome by A20 protects against arthritis. Nature 512: 69–73 25043000 10.1038/nature13322 PMC4126806

[emmm201810248-bib-0143] Vande Walle L , Lamkanfi M (2016) Pyroptosis. Curr Biol 26: R568–R572 27404251 10.1016/j.cub.2016.02.019

[emmm201810248-bib-0144] Venegas C , Kumar S , Franklin BS , Dierkes T , Brinkschulte R , Tejera D , Vieira‐Saecker A , Schwartz S , Santarelli F , Kummer MP *et al* (2017) Microglia‐derived ASC specks cross‐seed amyloid‐β in Alzheimer's disease. Nature 552: 355–361 29293211 10.1038/nature25158

[emmm201810248-bib-0145] Voet S , Mc Guire C , Hagemeyer N , Martens A , Schroeder A , Wieghofer P , Daems C , Staszewski O , Walle LV , Jordao MJC *et al* (2018) A20 critically controls microglia activation and inhibits inflammasome‐dependent neuroinflammation. Nat Commun 9: 2036 29789522 10.1038/s41467-018-04376-5 PMC5964249

[emmm201810248-bib-0146] Wallisch JS , Simon DW , Bayır H , Bell MJ , Kochanek PM , Clark RSB (2017) Cerebrospinal fluid NLRP3 is increased after severe traumatic brain injury in infants and children. Neurocrit Care 27: 44–50 28181102 10.1007/s12028-017-0378-7 PMC5680164

[emmm201810248-bib-0147] Walsh JG , Muruve DA , Power C (2014) Inflammasomes in the CNS. Nat Rev Neurosci 15: 84–97 24399084 10.1038/nrn3638

[emmm201810248-bib-0148] Wang W , Nguyen LTT , Burlak C , Chegini F , Guo F , Chataway T , Ju S , Fisher OS , Miller DW , Datta D *et al* (2016) Caspase‐1 causes truncation and aggregation of the Parkinson's disease‐associated protein α‐synuclein. Proc Natl Acad Sci USA 113: 9587–9592 27482083 10.1073/pnas.1610099113 PMC5003239

[emmm201810248-bib-0149] Wellington CL , Ellerby LM , Hackam AS , Margolis RL , Trifiro MA , Singaraja R , McCutcheon K , Salvesen GS , Propp SS , Bromm M *et al* (1998) Caspase cleavage of gene products associated with triplet expansion disorders generates truncated fragments containing the polyglutamine tract. J Biol Chem 273: 9158–9167 9535906 10.1074/jbc.273.15.9158

[emmm201810248-bib-0150] Wheeler RD , Boutin H , Touzani O , Luheshi GN , Takeda K , Rothwell NJ (2003) No role for interleukin‐18 in acute murine stroke‐induced brain injury. J Cereb Blood Flow Metab 23: 531–535 12771567 10.1097/01.WCB.0000059587.71206.BA

[emmm201810248-bib-0151] Wu P‐J , Hung Y‐F , Liu H‐Y , Hsueh Y‐P (2017) Deletion of the inflammasome sensor Aim2 mitigates Aβ deposition and microglial activation but increases inflammatory cytokine expression in an alzheimer disease mouse model. NeuroImmunoModulation 24: 29–39 28618410 10.1159/000477092

[emmm201810248-bib-0152] Xiao J , Wang C , Yao J‐C , Alippe Y , Xu C , Kress D , Civitelli R , Abu‐Amer Y , Kanneganti T‐D , Link DC *et al* (2018) Gasdermin D mediates the pathogenesis of neonatal‐onset multisystem inflammatory disease in mice. PLoS Biol 16: e3000047 30388107 10.1371/journal.pbio.3000047 PMC6235378

[emmm201810248-bib-0153] Xu H , Yang J , Gao W , Li L , Li P , Zhang L , Gong Y‐N , Peng X , Xi JJ , Chen S *et al* (2014) Innate immune sensing of bacterial modifications of Rho GTPases by the Pyrin inflammasome. Nature 513: 237–241 24919149 10.1038/nature13449

[emmm201810248-bib-0154] Xu B , Jiang M , Chu Y , Wang W , Chen D , Li X , Zhang Z , Zhang D , Fan D , Nie Y *et al* (2017) Gasdermin D plays a key role as a pyroptosis executor of non‐alcoholic steatohepatitis in humans and mice. J Hepatol 68: 773–782 29273476 10.1016/j.jhep.2017.11.040

[emmm201810248-bib-0155] Xu X , Yin D , Ren H , Gao W , Li F , Sun D , Wu Y , Zhou S , Lyu L , Yang M *et al* (2018) Selective NLRP3 inflammasome inhibitor reduces neuroinflammation and improves long‐term neurological outcomes in a murine model of traumatic brain injury. Neurobiol Dis 117: 15–27 29859317 10.1016/j.nbd.2018.05.016

[emmm201810248-bib-0156] Yan Y , Jiang W , Liu L , Wang X , Ding C , Tian Z , Zhou R (2015) Dopamine controls systemic inflammation through inhibition of NLRP3 inflammasome. Cell 160: 62–73 25594175 10.1016/j.cell.2014.11.047

[emmm201810248-bib-0157] Yang F , Wang Z , Wei X , Han H , Meng X , Zhang Y , Shi W , Li F , Xin T , Pang Q *et al* (2014) NLRP3 deficiency ameliorates neurovascular damage in experimental ischemic stroke. J Cereb Blood Flow Metab 34: 660–667 24424382 10.1038/jcbfm.2013.242 PMC3982086

[emmm201810248-bib-0158] Yang J , Liu Z , Xiao TS (2017) Post‐translational regulation of inflammasomes. Cell Mol Immunol 14: 65–79 27345727 10.1038/cmi.2016.29 PMC5214939

[emmm201810248-bib-0159] Yatsiv I , Morganti‐Kossmann MC , Perez D , Dinarello CA , Novick D , Rubinstein M , Otto VI , Rancan M , Kossmann T , Redaelli CA *et al* (2002) Elevated intracranial IL‐18 in humans and mice after traumatic brain injury and evidence of neuroprotective effects of IL‐18‐binding protein after experimental closed head injury. J Cereb Blood Flow Metab 22: 971–978 12172382 10.1097/00004647-200208000-00008

[emmm201810248-bib-0160] Youm Y‐H , Nguyen KY , Grant RW , Goldberg EL , Bodogai M , Kim D , D'Agostino D , Planavsky N , Lupfer C , Kanneganti TD *et al* (2015) The ketone metabolite β‐hydroxybutyrate blocks NLRP3 inflammasome‐mediated inflammatory disease. Nat Med 21: 263–269 25686106 10.1038/nm.3804 PMC4352123

[emmm201810248-bib-0161] Yuan B , Shen H , Lin L , Su T , Zhong S , Yang Z (2015) Recombinant adenovirus encoding NLRP3 RNAi attenuate inflammation and brain injury after intracerebral hemorrhage. J Neuroimmunol 287: 71–75 26439964 10.1016/j.jneuroim.2015.08.002

[emmm201810248-bib-0162] Yuan J , Amin P , Ofengeim D (2019) Necroptosis and RIPK1‐mediated neuroinflammation in CNS diseases. Nat Rev Neurosci 20: 19–33 30467385 10.1038/s41583-018-0093-1 PMC6342007

[emmm201810248-bib-0163] Zendedel A , Johann S , Mehrabi S , Joghataei M , Hassanzadeh G , Kipp M , Beyer C (2016) Activation and regulation of NLRP3 inflammasome by intrathecal application of SDF‐1a in a spinal cord injury model. Mol Neurobiol 53: 3063–3075 25972240 10.1007/s12035-015-9203-5

[emmm201810248-bib-0164] Zhang S , Tang M , Luo H , Shi C , Xu Y (2017) Necroptosis in neurodegenerative diseases: a potential therapeutic target. Cell Death Dis 8: e2905 28661482 10.1038/cddis.2017.286 PMC5520937

[emmm201810248-bib-0165] Zhou Y , Lu M , Du R‐H , Qiao C , Jiang C‐Y , Zhang K‐Z , Ding J‐H , Hu G (2016) MicroRNA‐7 targets Nod‐like receptor protein 3 inflammasome to modulate neuroinflammation in the pathogenesis of Parkinson's disease. Mol Neurodegener 11: 28 27084336 10.1186/s13024-016-0094-3 PMC4833896

